# Unravelling the Epitaxial Growth Mechanism of Hexagonal and Nanoporous Boron Nitride: A First‐Principles Microkinetic Model

**DOI:** 10.1002/smll.202405404

**Published:** 2025-01-05

**Authors:** Anthony J. R. Payne, Neubi F. Xavier Jr, Anton Tamtögl, Marco Sacchi

**Affiliations:** ^1^ School of Chemistry and Chemical Engineering University of Surrey GU2 7XH Guildford UK; ^2^ Institute of Experimental Physics Graz University of Technology Graz 8010 Austria

**Keywords:** chemical vapor deposition, hexagonal boron nitride, microkinetics, nanostructures, on surface synthesis, precursors, self‐assembly

## Abstract

Understanding the chemical and physical mechanisms at play in 2D materials growth is critical for effective process development of methods such as chemical vapor deposition (CVD) as a toolbox for processing more complex nanostructures and 2D materials. A combination of density functional theory and microkinetic modeling is employed to comprehensively investigate the reaction mechanism governing the epitaxial growth of hexagonal boron nitride (hBN) on Ru(0001) from borazine. This analysis encompasses four key stages prior to the formation of the complete hBN overlayer: (i) adsorption, diffusion and deprotonation of borazine, (ii) dimerization and microkinetic modeling (iii) stability of larger borazine polymers and (iv) formation of nanoporous intermediates. In doing so, the exact deprotonation sequence is followed for the first time, illustrating its crucial role for the formation of nanostructures. These findings not only provide insights into the epitaxial growth of hBN and the stability of intermediate overlayers, which are strongly dependent on surface temperature and the amount of precursor exposures, they offer also crucial guidance for producing high‐quality hBN monolayers with regular patterns or functionalisation. Importantly, these results align with experimental data and provide a detailed model which explains temperature‐dependent, in‐situ surface measurements during hBN growth on Ru and other substrates.

## Graphical abstract

This study explores the epitaxial growth of hexagonal boron nitride (hBN) on Ru(0001) using borazine, combining density functional theory and microkinetic modeling. Key stages include borazine adsorption, diffusion, deprotonation, dimerization, and nanoporous intermediate formation. The critical deprotonation sequence shapes nanostructures, offering insights into fabricating high‐quality hBN monolayers with tailored properties, supported by experimental validation.

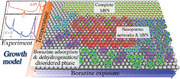

## 1 Introduction

2D hexagonal boron nitride (hBN) has attracted significant attention due to its unique properties and potential applications in various fields,^[^ ^1^ ^]^ including optics,^[^ ^2^ ^]^ catalysis,^[^ ^3^ ^]^ and absorption of pollutants.^[^ ^4^ ^]^ The performance of hBN in electrical applications is closely tied to the quality of the hBN sheet used for the device fabrication.^[^ ^5^, ^6^ ^]^ Other boron nitride based 2D allotropes containing four to eight‐membered rings instead of the six‐membered rings seen in hBN have been proposed theoretically,^[^ ^7^ ^]^ and extensive research focuses on enhancing hBN for applications in microelectronics by adding desirable properties via defects,^[^ ^8^, ^9^, ^10^, ^11^, ^12^, ^13^, ^14^, ^15^ ^]^ dopants,^[^ ^16^, ^17^, ^18^, ^19^, ^20^, ^21^, ^22^, ^23^, ^24^, ^25^, ^26^, ^27^, ^28^, ^29^, ^30^, ^31^, ^32^ ^]^ and chemical or physical functionalization.^[^ ^17^, ^33^, ^34^, ^35^, ^36^, ^37^, ^38^, ^39^, ^40^, ^41^, ^42^ ^]^ However, inhomogeneous dopant distribution can impact the properties of hBN,^[^ ^43^, ^44^, ^45^ ^]^ and therefore efforts have been made to control dopants' spatial distribution in hBN and other 2D materials.^[^ ^46^, ^47^, ^48^ ^]^

The chemical bond in hBN consists of sp^2^ hybridised B‐N covalent bonds. Due to the inherent electronegativity difference between the two elements (with Pauling electronegativities 2.04 and 3.04 for B and N, respectively),^[^ ^49^ ^]^ the nitrogen atoms exhibit a higher electron affinity and accept a greater share of the electron density in the covalent bonds, leading to a partial negative charge on the nitrogen atom and a corresponding partial positive charge on the boron atom. The distinct charge separation and localization of electron density contribute to the insulating nature of hBN with a wide bandgap of 6 eV.^[^ ^50^ ^]^ Moreover, the covalent sp^2^ hybridisation leads to high thermal stability,^[^ ^51^, ^52^ ^]^ and novel magnetic properties.^[^ ^22^ ^]^ Bulk and bilayer hBN have an AA' ground state stacking arrangement, where adjacent layers have alternating B and N atoms.^[^ ^53^, ^54^ ^]^

The controlled growth of hBN has been realised on several transition‐metal substrates, including Cu(111), Ni(111), Pt(111), Fe(110), and Ru(0001) via chemical vapor deposition (CVD).^[^ ^55^, ^56^, ^57^, ^58^ ^]^ The process typically employs dosing a B‐N‐containing gas precursor, such as ammonia borane (H_3_NBH_3_) or borazine (B_3_N_3_H_6_) onto the hot metal substrate at temperatures above 1000 K. Under such conditions, the borazine precursor is expected to decompose on the metal surface and form hBN sheets. However, high‐temperature CVD can also yield domains of borophene (a crystalline monolayer of boron),^[^ ^59^, ^60^ ^]^ borides,^[^ ^61^ ^]^ and the growth of multilayers from B and N reservoirs in the substrate.^[^ ^62^ ^]^ Importantly, recent studies at temperatures below the usual growth temperatures illustrated that additional phases form, such as nanostructures prior to the hBN formation^[^ ^63^ ^]^ or rotated phases of hBN.^[^ ^64^ ^]^

Hence, a detailed understanding of the hBN growth mechanism itself is necessary to improve the quality of hBN sheets and develop new boron nitride‐based materials. In light of recent findings about porous nanographene and graphene nanoribbons synthesis from a single molecular precursor,^[^ ^65^ ^]^ it appears likely that similar experimental protocols can be developed for the synthesis of nanoporous hBN structures. In particular, no method has been proposed to introduce defects, dopants, and functionalization in hBN with a regular spatial distribution. In contrast to other studies, we simulate the entire process from single borazine adsorption to diffusion, dehydrogenation and dimerization up to the formation of nanoporous structures together with a microkinetic model for the different phases. We follow a recent in‐situ hBN growth study, where a (3 × 3) nanostructure during the growth of hBN on Ru(0001) at temperatures between 750 and 880 K was identified.^[^ ^63^ ^]^ We combine Density Functional Theory (DFT) Calculations of geometries and transition states with microkinetic modelling to gain a comprehensive understanding of the hBN growth mechanism at temperatures below 900 K. Specifically we provide insight into two key questions:

- What is the nature of intermediate structures, and how / why do they depend on temperature?
- What is the role of precursor hydrogens in the growth mechanism?

Evidence for the presence of hydrogen during hBN growth was originally reported in pioneering work from the 1960s, where pyrolysis of borazine resulted in a BNH polymer at temperatures around 700 K.^[^ ^66^ ^]^ However, due to the difficulty to detect hydrogen atoms attached to adsorbed precursor molecules, an exact description of the deprotonation sequence is missing.

More recently, Scanning Tunnelling Microscopy (STM) images of hBN grown on Ru(0001) at temperatures between 700 and 900 K have shown that hBN islands are hydrogen terminated,^[^ ^67^ ^]^ with similar observations made when using Rh(111)^[^ ^68^ ^]^ and Ir(111)^[^ ^69^ ^]^ substrates, at temperatures below 900 K. The presence of hydrogen‐terminated hBN edges has been exploited to introduce GeO_2_ sites by dehydrogenating hBN on Cu foil at temperatures between 600 and 900 °C.^[^ ^70^ ^]^ Exposure of borazine to Ni at temperatures of 400 °C results in the formation of a borazine polymer with a rough surface morphology and hBN particles, with the crystallinity improved after annealing to 1000 °C to facilitate further dehydrogenation,^[^ ^71^ ^]^ with similar observations made for Cu.^[^ ^72^ ^]^ The morphology and rate of hBN growth are sensitive to the introduction of H_2_ along with the borazine precursor during CVD and it has been proposed that the diffusion of precursor fragments is affected by the abundance of hydrogen.^[^ ^73^ ^]^ Throughout CVD at temperatures below 1000 K, a proportion of the hydrogen atoms remain bound to the adsorbed precursor, as shown in thermal desorption mass spectroscopy experiments on Pt and Ru surfaces,^[^ ^74^ ^]^ with similar observations on Rh(111).^[^ ^75^ ^]^ During such low‐temperature CVD, partial dehydrogenation may lead to separation between precursor molecules, leading to the formation of intermediates that include nanoporous structures. Theoretical work employing DFT and molecular dynamics has been used to investigate hBN growth mechanisms,^[^ ^76^, ^77^, ^78^, ^79^ ^]^ however, these works did not consider the effect of hydrogen atoms on hBN growth at low temperatures, while a recent study on CVD of hBN from ammonia borane as a precursor, using DFT and *ab initio* molecular dynamics at 1500 K, has highlighted the importance of the involvement of H atoms in the epitaxial growth of hBN on Ni(111) surfaces, which occurs through a stable BNH_2_ species.^[^ ^80^ ^]^

Given the experimental evidence for partial dehydrogenation of hBN precursors, we begin our investigation by first considering the behavior of an isolated borazine molecule on Ru(0001) before investigating dimerization and finally considering larger borazine‐based polymer structures.

## 2 Methodology

DFT was used in spin‐polarized electronic calculations completed using the CASTEP code.^[^ ^81^ ^]^ The exchange‐correlation potential was parameterized using the Perdew–Burke–Ernzerhof (PBE) functional.^[^ ^82^ ^]^ Vanderbilt ultrasoft pseudopotentials^[^ ^83^ ^]^ were employed with a cutoff energy of 400 eV for the plane wave basis set. Dispersion corrections were accounted for using the Tkatchenko and Scheffler method.^[^ ^84^ ^]^ Integration over the Brillouin zone was performed on a 4 × 4 × 1 or 2 × 2 × 1 *k*‐point grid for modeling (3 × 3) and (6 × 6) unit cells, respectively, generated by the Monkhorst–Pack method.^[^ ^85^ ^]^ The Ru(0001) surface was modeled using a five‐layer slab with the positions of the atoms in the top three layers of the Ru substrate and the adsorbates left unconstrained with an additional vacuum layer of 15 Å set to avoid spurious interactions between the periodically repeated images. Geometry optimizations were performed until the maximum force on each atom was less than 0.025 eV Å^−1^. The Linear and Quadratic Synchronous Transit (LST/QST) algorithm^[^ ^86^ ^]^ was used to identify transition states along the reaction pathways, with a force tolerance of 0.05 eV Å^−1^. The tolerance for energy convergence was set to 1×10^−6^ eV. In our previous work, this computational framework has been shown to replicate the B–N bond length and lattice parameter of hBN accurately.^[^ ^87^ ^]^

Vibrational states were calculated at the Γ‐point using partial Hessian vibrational analysis.^[^ ^92^ ^]^ Only the normal modes of the adsorbate and the top layer of the Ru(0001) substrate were considered (all other layers were fixed). The entropies and partition functions were estimated according to the lattice gas approach^[^ ^93^, ^94^ ^]^ with reaction rate coefficients calculated for chemisorption, surface reactions, subsurface diffusion and desorptions according to transition state theory. To avoid inconsistency, vibrational modes with values lower than 200 cm^−1^ were assumed to be 200 cm^−1^, as commonly adopted in the literature.^[^ ^95^, ^96^ ^]^ The equation *G* = *H* − *T* *S* was used to calculate the Gibbs free energy where *S* is the entropy and the enthalpy, *H*, comprises the DFT energy at 0 K, the zero‐point energy correction and thermal contributions at temperatures ranging between 25 and 1000 K. The difference between the free energies of the transition state and the initial state was used to estimate the Gibbs free energy of activation, Δ*G* ^‡^. In order to determine the surface coverage of species at different temperatures a set of ordinary differential equations (ODEs) were obtained for each species *i*:

(1) $$\frac{\partial \theta_{i}}{\partial t}=\sum_{\pm j}c_{i,\pm j}r_{\pm j}$$

where *c*_*i*, ±*j*_ is the concentration of species *i* in the elementary reaction where +*j* and −*j* are the forward and backward reactions, respectively. *r*_±*j*_ is the rate of the respective reaction. The MKMCXX code^[^ ^97^ ^]^ was then used to solve the ODEs at steady state ($\frac{\partial \theta_{i}}{\partial t}=0$) using the stiff solver method, similar to our previous work.^[^ ^95^, ^98^ ^]^ A pressure of 5 · 10^−8^ mbar was applied to conform to experimental conditions,^[^ ^63^ ^]^ with borazine initialized in the gas phase. Full details of the microkinetic model can be found in Section S2 (Supporting Information). Molecular diagrams were produced using VESTA.^[^ ^114^ ^]^

## 3 Results and Discussion

**Figure 1:**
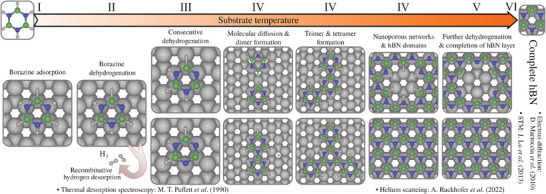
Overview of several steps upon chemical vapour deposition of hBN on Ru(0001) using borazine (B_3_H_6_N_3_) as a precursor gas. The onset of the individual steps is a function of the Ru substrate temperature with the regions I−VI. Experimental evidence for the hBN/Ru system is based on electron diffraction (D. Martoccia et al. (2010)^[^ ^88^ ^]^) and STM studies (J. Lu et al. (2013)^[^ ^89^ ^]^) of the complete hBN layer, while the nanoporous network prior to the completion of the hBN layer has been observed in helium scattering measurements (A. Ruckhofer et al. (2022)^[^ ^63^ ^]^). Finally, experimental information about dehydrogenation is only available in an indirect way via temperature programmed desorption measurements of H_2_ (M. T. Paffett et al. (1990)^[^ ^74^ ^]^) following borazine adsorption. Further experimental evidence comes from hBN growth on similar substrates, such as, e.g., individual and borazine dimers in STM measurements on Ag(111),^[^ ^90^ ^]^ or NEXAFS studies of borazine adsorption on Rh(111).^[^ ^91^ ^]^

To understand the physicochemical details of the hBN growth mechanism on Ru(0001), we follow the experimental sequence, as illustrated in the overview in **Figure** **1** together with corresponding experimental results for hBN/Ru(0001). We first establish the adsorption sites of the borazine precursor, followed by its diffusion across the surface, along with potential deprotonation and decomposition. Next, we explore the formation of borazine dimers and develop a microkinetic model. We then consider the formation of larger borazine polymer structures before discussing the formation of nanoporous structures. In each section, the influence of each process on the resulting structure is analyzed. Altogether it provides a detailed theoretical description of the individual steps and their dependence on experimental growth parameters, for the synthesis of hBN and nanoporous hBN structures.

### 3.1 The Adsorbed Borazine Monomer

The nature of the bonding between the hBN monolayer and a transition metal substrate has been well established as a balance between overall repulsive forces acting on N and attractive forces on B.^[^ ^99^ ^]^ N atoms occupy the site directly on top of the surface metal atoms, with B atoms occupying the FCC site.^[^ ^57^ ^]^ Furthermore, the B atom can occupy an HCP site with only slightly higher total energy. All other configurations are unstable, leading to a repulsive N‐metal interaction that outweighs the attractive B‐metal interaction.^[^ ^99^ ^]^ The selectivity of N atoms for the top site and the potential of B atoms to occupy two possible sites give rise to two highly favored arrangements of hBN on low‐index transition metal surfaces (N_TOP_B_HCP_ and N_TOP_B_FCC_) which have been observed as domains growing at a 30‐degree angle on Ni(111).^[^ ^100^ ^]^ In terms of experimental studies of borazine adsorption itself there exist, however, only very few studies,^[^ ^69^, ^101^, ^102^ ^]^ with the most recent being a high‐resolution STM study which illustrate the self‐assembly of intact borazines into a porous structure on Ag(111) at temperatures below 150 K.^[^ ^90^ ^]^

**Figure 2:**
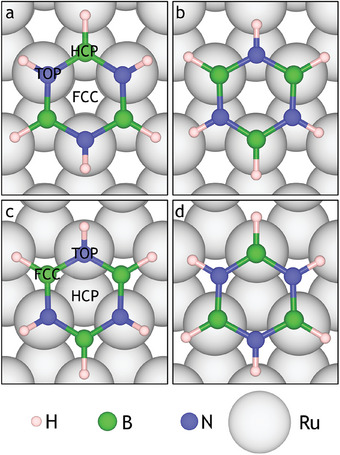
Adsorption sites of borazine on Ru(0001) show a clear trend for N_TOP_ being preferred over HCP and FCC sites. a) Borazine centered on the FCC site with N_TOP_ and B_HCP_ (*E*_ads_ = −4.08  eV) b) Borazine centered on the FCC site with N_HCP_ and B_TOP_ (*E*_ads_ = −1.29  eV) c) Borazine centered on the HCP site with N_TOP_ and B_FCC_ (*E*_ads_ = −4.00  eV) d) Borazine centered on the HCP site with N_FCC_ and B_TOP_ (*E*_ads_ = −1.32  eV). Energetically, only the adsorption configurations (a) and (c) are likely, with (a) being more favorable by 0.08 eV.

We considered the adsorption site of borazine on the Ru(0001) surface, as shown in **Figure** **2**. Similar to the complete hBN layer, significant energy differences arise depending on the position of the nitrogen atom. Adsorption energies *E*_ads_ of borazine were calculated using the formula:

(2) $$E_{\mathrm{ads}}=E_{\mathrm{AB}}-E_{A}-E_{B}$$

where *E*_AB_ is the energy of the adsorbed species, *E*_A_ and *E*_B_ are the energy of the dissociated species.

The most favorable adsorption site of borazine is with the N situated directly on top of the surface Ru atom with an *E*_ads_ of −4.08  eV. Adsorption of N onto the HCP and the FCC sites increases the *E*_ads_ to −1.29  and −1.32  eV, respectively. Such significant differences in *E*_ads_ show that N_TOP_ adsorption is greatly favored over N_HCP_ and N_FCC_ sites, therefore limiting the possible arrangements of borazine on Ru(0001) to the N_TOP_ site. With the N_TOP_ site occupied, B_HCP_ becomes the most favored B site, with a slightly lower *E*_ads_ of −4.08  eV compared to −4.00  eV in the case of B_FCC_. Thus, as shown in Figure 2a, borazine centered on the FCC site with the N_TOP_ B_HCP_ configuration is the most favorable adsorption site. The interactions with the Ru(0001) substrate are consistent for both borazine and hBN and limit the equilibrium positions of N and B atoms in each case. As borazine and hBN have limited favorable configurations, the likely configurations of the intermediate structures can be restricted to those with N_TOP_ and B_FCC_ or B_HCP_.

**Figure 3:**
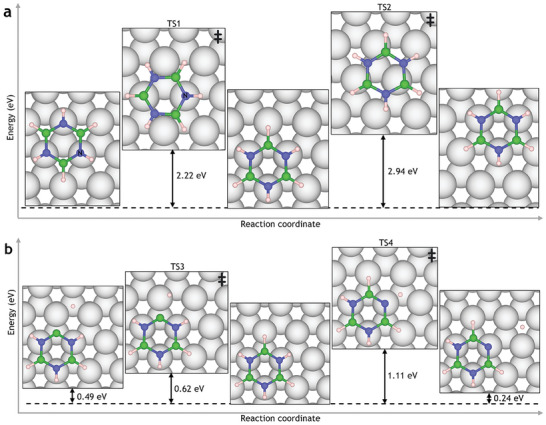
a) Diffusion routes of borazine on Ru(0001) illustrate that borazine moves by a pivoting motion alternating between HCP and FCC sites as it diffuses across the surface. Rotation and translation between FCC and HCP sites pivoting around a stationary N_TOP_ site (labeled N) exhibit an energy barrier of 2.22 eV (TS1), while translation between FCC sites without rotation exhibits an energy barrier of 2.94 eV (TS2). In terms of hydrogen removal from borazine, b) shows that B‐dehydrogenation is the kinetically favored product, whilst N‐dehydrogenation is thermodynamically favored. Borazine can dehydrogenate by breaking a B–H or N–H bond with energy barriers of 0.62 and 1.11 eV, respectively (TS3 and TS4). The borazine dehydrogenation pathways on Ru(0001) to produce an adsorbed B_3_N_3_H_5_ species show that the total change in energy (Δ*E*) of the reaction is +0.49 and +0.24 eV for B and N dehydrogenation, respectively, thus, the latter is thermodynamically favored.

Having established the allowed configurations of the borazine on Ru(0001), we next consider the diffusion of borazine between the HCP and FCC sites. It appears that once borazine adsorbs at an FCC site, the molecule is unlikely to rotate around its centre of mass, as this would move all the N atoms to an unfavorable site. Instead, borazine is likely to diffuse between HCP and FCC sites by rotation and translation occurring simultaneously. The transition states for these diffusion pathways were calculated and are displayed in **Figure** **3**a. The energy barrier to translate between FCC sites is 2.94 eV (TS2), where all of the atoms move simultaneously across the surface. Translation with rotation, however, has a lower energy barrier of 2.22 eV (TS1) and occurs by pivoting around a stationary nitrogen (labeled N in Figure 3). The pivoting motion is preferred by 0.72 eV as it allows one of the N atoms to maintain its position on top of the Ru, which minimizes repulsion from the surface. In contrast, in the case of direct translation, all nitrogen atoms pass through unfavorable sites, raising the energy of this transition state.

Despite the pivoting motion being preferred, the high barrier of 2.22 eV suggests that adsorbed borazine molecules are unlikely to diffuse at low temperatures. Furthermore, increasing the temperature stabilises the transition state TS1, lowering the Δ*G* ^‡^ to 1.88 eV at 900 K, allowing diffusion to occur more readily. The ability of borazine to pivot and diffuse across the Ru(0001) surface under growth conditions allows the molecules to approach one another and form dimers. The pivoting diffusion mechanism is particularly relevant for their subsequent polymerization, especially at low surface coverages and dosages. The constrained arrangements of borazine on the surface, combined with restricted diffusion routes, limit the possible approaches of two borazine molecules toward each other, thus influencing polymerization, as discussed in Section 3.3.

The first step for borazine polymerization requires the removal of hydrogen atoms from a borazine molecule. To compare borazine species with different stages of partial dehydrogenation, we can define a reaction energy *E*_r_:

(3) $$E_{r}=E_{\mathrm{tot}}\left(x\right)+nE_{\mathrm{tot}}\left(H_{\mathrm{ads}}\right)-E_{\mathrm{tot}}\left(Ru+mBZ\right)$$

where *E*_tot_(*x*) is the total energy of the borazine species, *n* is the number of hydrogen atoms removed to create the borazine species, *E*_tot_(*H*_ads_) is the total energy of chemisorbed hydrogen, and *E*_tot_(*Ru* + *mBZ*) is the total energy of the isolated Ru surface and *m* isolated borazine molecules.

The dehydrogenation of borazine occurs from either B or N atoms with transition state barriers of 0.619 and 1.106 eV, respectively, and is shown in Figure 3b. N‐dehydrogenation has a higher activation energy barrier but lower reaction energy; thus, it is the thermodynamically favored product of the reaction, whereas B‐dehydrogenation is kinetically favored. N, a more electronegative atom than B, has stronger N–H bonds in borazine, as shown by bond lengths of 1.02 and 1.26 Å for N–H and B–H bonds, respectively. Therefore, the barrier to breaking N–H bonds is higher than that for B–H bonds.

N dehydrogenation yields a product that is notably more thermodynamically stable compared to B‐dehydrogenation, with a total energy difference of 0.14 eV. Upon losing an N–H bond, the nitrogen has reduced connectivity. Therefore, N bonds more strongly to the adjacent B atoms, with bond lengths changing from 1.48 to 1.43 Å, accompanied by a marginal reduction in negative charge on the hydrogenated N from −0.740  to −0.620  e. The reduction in partial negative charge reduces the electrostatic repulsion between the N and the Ru, stabilizing the structure and resulting in a lower *E*_r_ of −3.86  eV. B‐dehydrogenation allows the B atom to bend further out of the plane toward the surface, where it can make a weak bond to the surface with a Mulliken population of 0.55 e. However, by moving out of the plane, the sp^2^ bonds to N have weakened, thus resulting in an *E*_r_ of −3.72 eV. Overall, N‐dehydrogenation is preferred due to the reduced electrostatic repulsion between N and Ru.

**Figure 4:**
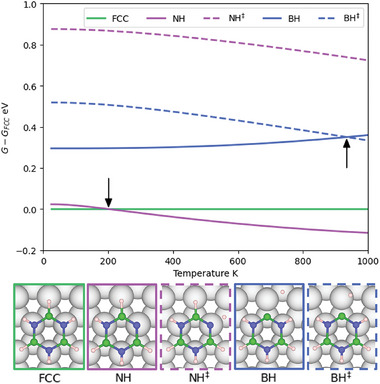
Plot of *G* values for dehydrogenated borazine geometries as shown in the lower panels and transition states relative to *G* of borazine at the FCC site. At around 200 K, the relative *G* for N‐dehydrogenation becomes negative and thus spontaneous above this temperature. Furthermore, the relative *G* curves for the B dehydrogenation geometry and its transition state intersect at 935 K. Hence, B‐dehydrogenation will become barrierless at temperatures >900 K.

**Figure** **4** shows how the Gibbs free energy of the dehydrogenated structures (*G*) and transition states (*G* ^‡^) varies with the temperature relative to the *G* of borazine in the FCC site. A plot of the change in G with temperature can be seen in Figure S1 (Supporting Information). The free energy of N‐dehydrogenated borazine decreases as the temperature increases due to an increase in vibrational entropy, with higher temperatures further extending the distances between atoms and minimalizing electrostatic repulsion. The stabilizing effect results in N‐dehydrogenation becoming the thermodynamically favorable product at temperatures >200 K. Conversely, the B‐dehydrogenated structure becomes increasingly unfavorable thermodynamically due to the B atom moving away from the optimum Ru bonding site, reducing attractive interactions.

At 935 K, the relative *G* ^‡^ for B‐dehydrogenation becomes similar to the relative *G* for the B‐dehydrogenated structure, converging to 0.35 eV at 950 K. The relative *G* ^‡^ for the N‐dehydrogenated isomer lowers slightly with temperature, resulting in a relative *G* ^‡^ of 0.75 eV at 950 K. Given the relative *G* and *G* ^‡^ for the dehydrogenated structures, we expect N‐dehydrogenation to be kinetically limited, and B‐dehydrogenation to be thermodynamically limited.

In addition to the direct dehydrogenation of B and N (from borazine to an available substrate site), we also considered the transfer of H between adjacent under‐coordinated B and N atoms, as shown in Figure S2 (Supporting Information). The H transfer occurs with a barrier of 2.21 eV from N to B, similar to the barrier for borazine diffusion. Therefore, hydrogen transfer between nitrogen and boron atoms becomes kinetically viable only at elevated temperatures, with a preference for dehydrogenation.

**Figure 5:**
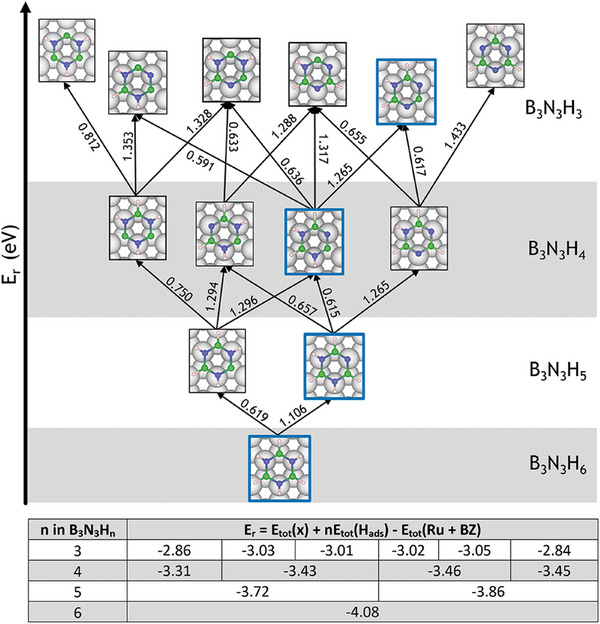
Reaction and transition state energies for consecutive dehydrogenation from the complete borazine molecule to B_3_N_3_H_3_ with the most stable configuration for each stoichiometry highlighted with a blue border. The transition state energies for B‐dehydrogenation are consistently lower (average 0.66 eV) compared to N‐dehydrogenation (average 1.32 eV). The lower table shows the corresponding reaction energies, which increase with increasing dehydrogenation. The arrows indicate the reaction pathway labeled with the energy of the corresponding transition state for dehydrogenation.

Having compared the first dehydrogenation step from B and N, we now consider a second dehydrogenation step. As shown in **Figure** **5** there are four possible structures with the chemical formula B_3_N_3_H_4_, with the most favorable structure occurring when two H are dehydrogenated from adjacent N and B atoms with an *E*_r_ of −3.46  eV. The adjacent deprotonation structure is only slightly favorable compared to dehydrogenating from two N (*E*_r_ of −3.45  eV) and dehydrogenation from opposite N and B atoms in the ring (*E*_r_ of −3.43  eV). Similarly to single dehydrogenation, N now has a charge of 0.61  e, and B makes a weak bond to the surface Ru. Stronger N–B binding of dehydrogenated atoms leads to an increase of charge on B from +0.08 e to +0.13 e, thus increasing the electrostatic attraction between B and the surface. Dehydrogenation of adjacent B and N sites reduces the N‐Ru repulsive interaction and increases the B‐Ru attractive interaction; therefore, losing adjacent H atoms leads to a more stable configuration. The dehydration of a B and N atom on opposite sides of the borazine molecule is subject to similar N‐Ru repulsive and B‐Ru attractive interactions. However, the stabilizing effects are lessened due to the absence of the reinforced N–B binding characteristic of adjacent dehydrogenated atoms, with the charge on the B atom increasing by only +0.02 e. Consequently, deprotonation from opposite B and N sites results in a higher energy isomer compared to adjacent B and N sites. Other dehydrogenated isomers entail the removal of hydrogen from either N or B atoms, which serves to alleviate repulsion or enhance attraction rather than producing a synergistic effect.

Removing a third hydrogen from borazine leads to six possible structures, as shown in Figure 5. The energy differences between these B_3_N_3_H_3_ structures can be explained by following similar trends as the B_3_N_3_H_4_ structures, where the structures with more adjacent dehydrogenations have a lower reaction energy and are more stable. The isomers with the lowest energy occur when three adjacent H atoms from one side of the molecule with two N and one B site are deprotonated. Isomers with the highest energies are observed when hydrogen atoms are dehydrogenated from the same element, either B or N atoms, without any adjacent dehydrogenation. We can use the trends established here to understand how larger borazine polymer structures will likely dehydrogenate.

For each B_3_N_3_H_n_ isomer, the dehydrogenation from B atoms is kinetically favorable compared to N atoms, with average barriers of 0.66 eV and 1.32 eV, respectively. We can identify potential reaction pathways by considering the sequential dehydrogenation from B_3_N_3_H_6_ to B_3_N_3_H_3_ through the lowest‐energy isomer of each stoichiometry. The most stable B_3_N_3_H_5_ isomer can be produced by overcoming an energy barrier of 1.106 eV. Overcoming total energy barriers of 1.721 and 2.986 eV leads to the formation of the most stable isomers with B_3_N_3_H_4_ and B_3_N_3_H_3_stoichiometry, respectively, corresponding to sequential dehydrogenation of adjacent N, B and N atoms. Alternative pathways include sequential dehydrogenation of two N atoms before the B‐atom in between is dehydrogenated with a total energy barrier of 2.988 eV. In each case, dehydrogenation is accompanied by an increase in energy due to the loss of connectivity. In subsection 3.2, we discuss the mechanism for borazine polymerization where the B–N bond formation bond provides a thermodynamic driving force for the reaction.

**Figure 6:**
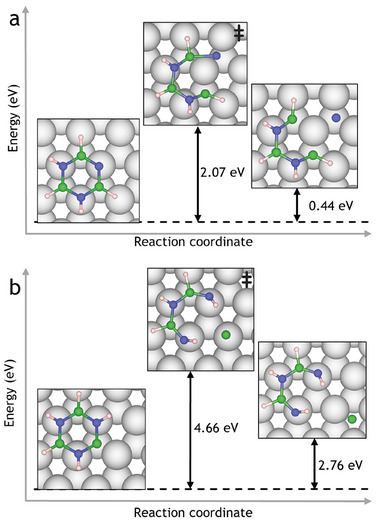
Decomposition of dehydrogenated borazine (B_3_N_3_H_5_) on Ru(0001) by breaking of a B–N bond to produce adatoms and an incomplete ring. a) N‐dehydrogenated borazine is decomposed to produce a nitrogen adatom on the surface. b) breaking B–N bonds from a B‐dehydrogenated borazine to produce a B surface adatom.

In addition to borazine dehydrogenation and diffusion, the decomposition of borazine to constituent atoms was also explored. Decomposition involves breaking B–N bonds in the borazine ring to liberate adatoms onto the Ru(0001) surface, with results shown in **Figure** **6** and Figure S3 (Supporting Information). Given the high barriers involved, we expect the borazine ring to break only at high temperatures after dehydrogenation. The solubility of B and N atoms experimentally depends on the metal, with both species showing a tendency for subsurface diffusion into Fe,^[^ ^103^ ^]^ N shows low solubility in Ni^[^ ^104^ ^]^ and only B is soluble in Cu.^[^ ^105^ ^]^ Via DFT it has been shown that N has a much greater barrier to subsurface diffusion than B on Pt(110),^[^ ^106^ ^]^ and that a low solubility of N in Ni exists. The possibility of adatom diffusion into the bulk of Ru was also explored and is shown in the Supporting Information. Diffusion of a B atom was shown to have a transition state barrier of 2.99 eV and a Δ*E* of +0.31 eV to move from the surface underneath the first layer. In contrast, N diffusion would result in a prohibitively high Δ*E* of +3.47 eV. Therefore, the solubilities of B and N are similar to the Cu case, where only B is soluble. As the reaction barriers are much greater than that of dehydrogenating borazine molecules, we can expect the surface to be dominated by borazine‐based structures at moderate temperatures. These findings are consistent with DFT simulations on Cu,^[^ ^107^ ^]^ and simulations of ammonia borane on Ni(111) at 1500 K using DFT combined with Ab Initio Molecular Dynamics,^[^ ^80^ ^]^ where the B–N bond remains intact.

In summary, our results on borazine adsorption, diffusion, and consecutive dehydrogenation indicate that only specific orientations are favorable (N_top_ with B_FCC_ or B_HCP_). These orientations correspond to the domains of hBN and restrict the potential structures of polymer intermediates. Furthermore, diffusion of borazine is likely to occur by pivoting around a stationary N, which limits the possible ways two molecules can approach each other. By investigating dehydrogenation, we have found that N‐dehydrogenation is thermodynamically favorable compared to B‐dehydrogenation, with preferntial loss of adjacent hydrogens. However, N‐dehydrogenation has a higher barrier than B‐dehydrogenation. Therefore, the hBN growth mechanism is complex with thermodynamic and kinetic contributions, which are highly dependent on the surface temperature. Using this information, we can now investigate how two borazine molecules dimerise.

### 3.2 Dimerization of Borazine

**Figure 7:**
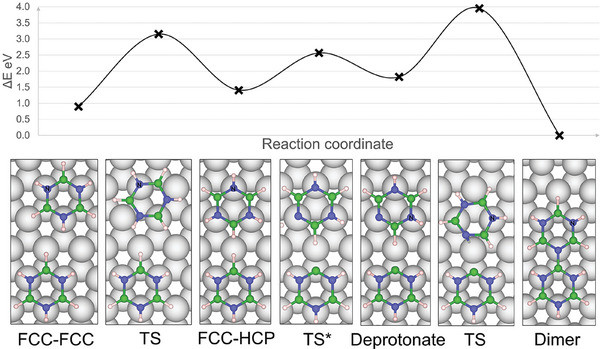
The dimerization mechanism of borazine on Ru(0001), from left to right: Neighboring borazines must first diffuse to adjacent FCC and HCP sites with an energy barrier of 2.2 eV. Then, dehydrogenation occurs with relatively low energy barriers of 0.64 and 1.17 eV for B and N atoms, respectively. Finally, one borazine molecule diffuses so close that a B–N bond is formed between the molecules to create a dimer. The stationary N_TOP_ sites are labeled N in each diffusion step.

Having established the diffusion pathways of borazine and its dehydrogenation mechanism, we can now consider how two borazine molecules can dimerize on the Ru(0001) surface. For dimerization to occur, at least one H atom from each molecule must be removed. The dimerization mechanism is illustrated in **Figure** **7**.

Starting from the left‐most figure, one of the molecules diffuses between FCC sites and HCP sites by pivoting around a stationary nitrogen (discussed in Section 3.1) with energy barriers of 2.2 eV (TS1‐3), eventually reaching the HCP adjacent to a molecule in the FCC site (labeled ‘FCC‐HCP’ in Figure 7). The next dehydrogenation from B and N occurs with barriers of 0.64 and 1.17 eV, respectively, and the hydrogen atom diffuses across the surface away from the borazine molecules. Surface hydrogen atoms have an *E*_ads_ of −0.73 eV with a diffusion barrier of 0.15 eV to move between FCC and HCP sites and will eventually undergo associative desorption as H_2_.^[^ ^74^ ^]^ Finally, one of the borazines pivots around a stationary nitrogen and moves toward the second borazine to create a new B–N bond, thus, completing the formation of the dimer with all N atoms at a top site and all B at the HCP sites. The overall reaction is thermodynamically favorable at 0 K with an Δ*E* of –0.91 eV, and the highest barrier (2.31 eV) in the dimerization process is the diffusion of borazine from the FCC site to the HCP site.

**Figure 8:**
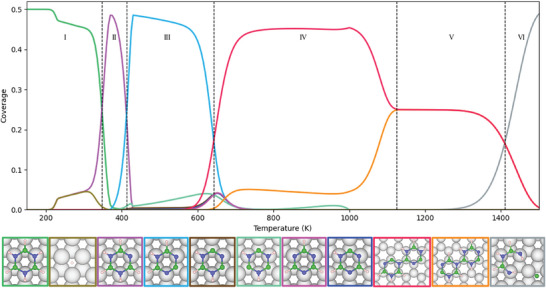
Illustration of the temperature dependent variation for the abundance of different borazine species and structures according to the microkinetic model. The different species and structures can be separated into 6 temperature regimes. A low temperature region I II as the temperature is increased to around 400 K, where a B_3_N_3_H_5_ species with dehydrogenated N dominates together accompanied by recombinative desorption of H_2_. In region III IV, dimerization gives rise to structures which are likely to lead to nanopore formation prior to hBN synthesis. Eventually, at around 1000 K, the selectivity of the dimer and polymer structures begins to decrease, and at temperatures >1000 K, domains of hBN and the nanopore are expected to form concurrently. In V at temperatures >1100 K hBN is expected to form directly, without the nanopore structure. Finally, at temperatures around 1400 K (VI), B–N bonds can be readily broken, resulting in the decomposition of the borazine ring and subsurface diffusion of individual B atoms.

Given the complexity of borazine dehydrogenation, diffusion and dimerisation, we performed microkinetic modeling of the overall epitaxial growth by linking individual reactions. The microkinetic model in **Figure** **8** shows how the coverage of different B_3_N_3_H_n_ structures changes with temperature. A total of 20 unique reactions were considered and are shown in tables S1 to S3 (Supporting Information). As the difference in *E*_ads_ between borazine HCP and FCC sites is low (see Figure S4, Supporting Information), the FCC and HCP sites were treated as equivalent; further details of the model are discussed in section S2 (Supporting Information). The intact borazine dominates the surface at lower temperatures (region I in Figure 8). At temperatures above 350 K, the concentration of N‐dehydrogenated B_3_N_3_H_5_ begins to increase and dominates the surface coverage. The concentration of excess hydrogen increases with N‐dehydrogenated B_3_N_3_H_5_ before dropping to 0 at 400 K due to the onset of recombinative desorption of H_2_. Desorption at 375 K coincides with the hydrogen peak in the thermal desorption spectrum.^[^ ^74^ ^]^ From 500 K onwards, a B_3_N_3_H_3_ species, with adjacent dehydrogenated B and N atoms, becomes the most abundant species together with other B_3_N_3_H_3_ molecules present at a much lower density (region III).

In region IV, sarting at 650 K, borazine molecules are able to move between FCC and HCP sites and dimerise following the mechanism shown in Figure 7. Given the analogous ring structures of borazine and benzene, we adopt the *ortho*, *meta*, and *para* terminology used for substituted benzene rings to describe the deprotonated isomers of borazine dimers (Figure S8, Supporting Information, illustrates the convention used). Due to the preferential dehydrogenation of adjacent hydrogen atoms, dimers with exposed *meta* and blocked *para* sites start to dominate the surface; such dimers lead to the formation of curved or C‐shaped polymer structures, as further discussed in Section 3.3 (e.g., Figure 10a). At temperatures lower than 700 K, surface structures are produced by random sequential adsorption, leading to disordered phases on the Ru(0001) surface.

**Figure 9:**
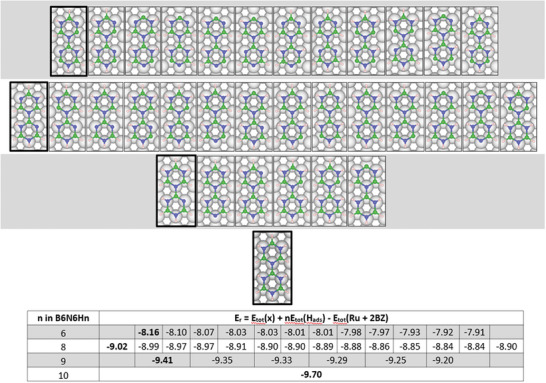
In analogy to single borazine molecules, the reaction energies for hBN dimers with different levels of dehydrogenation illustrate that N dehydrogenation is initially preferred, followed by dehydrogenation of adjacent B N atoms upon dehydrogenation of multiple hydrogen atoms. Furthermore, N dehydrogenation from the *ortho* position is favored, followed by dehydrogenation of opposite sites of the dimer.

**Figure 10:**
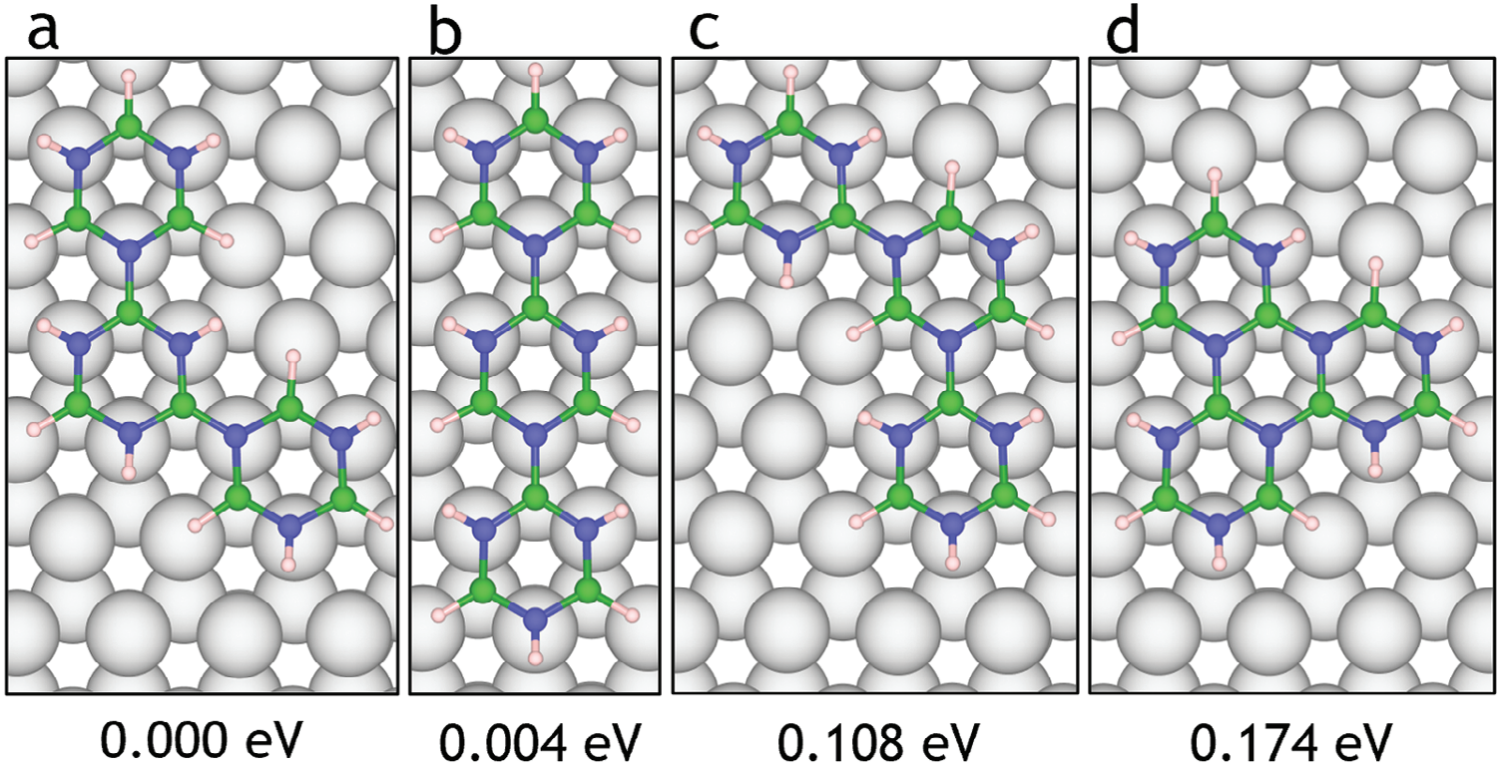
Relative energies for the formation of borazine trimers with different configurations and increasing energy from left to right. (a) and (c) illustrate “C‐shaped” trimer structures with the central borazine having B‐N bonds to outer molecules in the *meta* position. In (a), the central borazine is connected via B atoms, while in (c), the central atom is connected via N atoms. (b) illustrates a “line” structure where the central borazine is bonded through *para* positions, and (d) illustrates the formation of a cluster. A combination of (a) and (c) can lead to the formation of a nanopore, while continuing (b) would lead to long line structures as illustrated in Figure 12b.

Above 700 K, diffusion of borazine becomes possible and allows the creation of ordered structures. From 1000 K, dimers with exposed *para* sites start to dominate. These dimers are associated with the formation of linear superstructures (as discussed in Section 3.3) and are present in equal coverage with the C‐shaped structures at 1100 K. Such a behavior is expected since the dimerization mechanism to form C‐shaped and linear structures has a similar barrier. The dimer produced depends on the occurrence of B_3_N_3_H_n_ structures that form the constituents of each dimer.

At temperatures below 1100 K, B_3_N_3_H_n_ structures with adjacent dehydrogenations are thermodynamically favored, leading to the selective formation of C‐shaped structures. At temperatures above 1100 K, competing dehydrogenation reactions become more frequent; therefore, a larger variety of dehydrogenated borazine isomers are produced, any selectivity for a particular isomer is lost, and hBN domains are formed. Such formation of hBN domains is consistent with the typical synthesis of hBN on Ru(0001) from borazine at a temperature of 1050 K.^[^ ^57^ ^]^

Moreover, above 1400 K B‐N bonds are now able to break with the mechanism shown in Figure 6, giving rise to boron atoms diffusing into the substrate as described in Subsection 3.1. The temperature of ring breaking is consistent with the thermal degradation of hBN nanoplatelets,^[^ ^108^ ^]^ and hBN growth via atom formation and interstitial diffusion on Fe (1150 K),^[^ ^61^, ^103^ ^]^ Cu (1250 K),^[^ ^105^ ^]^ and Ni(111) (1400 K) substrates.^[^ ^62^ ^]^

The temperatures at which changes in surface coverage occur are consistent with temperature‐programmed X‐ray photoelectron spectroscopy measurements on Rh(111), where borazine predominates the surface coverage up to temperatures of approximately 200 K before initial deprotonation.^[^ ^91^ ^]^ Subsequently, deprotonated and disordered phases emerge at temperatures above approximately 350 K. Eventually, the surface is dominated by the formation of hBN at temperatures exceeding 900 K.

Given that dimerization and polymerization are controlled through a diffusion‐limited mechanism, the formation of an intermediate (3 × 3) network strongly depends on both substrate temperature and borazine exposure. The substrate temperature needs to be high enough to facilitate the diffusion of borazine molecules, while with increasing exposure, the excessive borazine density at the surface will impede the discussed diffusion and dimerization pathway, resulting in the creation of less ordered boron nitride structures. The latter is consistent with the disappearance of the (3 × 3) phase at higher borazine exposures in experiments.^[^ ^63^ ^]^

As the dimerization depends on the diffusion of borazine and dehydrogenated borazine species (B_3_N_3_H_n_) and both exhibit similar barriers for diffusion (see Figure S6, Supporting Information), we assume that the dimerization barrier of each species and each combination is sufficiently similar to be treated in the same way. We also note that the model considers only the initial bond‐breaking step in the decomposition of the borazine molecule, which results in high‐temperature hBN formation pathways similar to those explored in previous studies^[^ ^76^, ^78^, ^79^ ^]^ and pathways analogous to that of ammonia borane.^[^ ^80^ ^]^ Due to these assumptions, our model may overestimate the formation temperature of hBN and the temperature of borazine decomposition. Nevertheless, the microkinetic model accurately captures the temperature‐dependent coverage as observed in experiments, including the H_2_ desorption trends identified through thermal desorption studies^[^ ^74^ ^]^ and the emergence of an ordered (3 × 3) structure at temperatures exceeding 750 K during *in situ* diffraction studies.^[^ ^63^ ^]^ Furthermore, the model aligns with high‐temperature CVD observations where borazine decomposition leads to hBN formation.^[^ ^57^ ^]^ The excellent agreement between the model and experimental observations strongly supports its capacity to represent the behavior of borazine on Ru(0001) across a wide temperature range.

As shown in **Figure** **9**, similar to the single borazine molecule, different levels of dehydrogenation were considered for the dimers. Dehydrogenation from N continues to be thermodynamically favorable for the first dehydrogenation, with the *ortho* position being the most favorable, followed by the *para* and *meta* positions. The second dehydrogenation is thermodynamically favorable to occur from the *ortho* H on the opposite side, followed by dehydrogenation of 2 H closest to the bond as H_2_. When considering two dehydrogenations from each borazine ring in the dimer, the preferential dehydrogenation of adjacent hydrogen atoms continues with dehydrogenation on opposite sides of the dimer. The reason for this is much the same as for the isolated borazine; adjacent dehydrogenation increases the Ru‐B attraction and reduces the Ru‐N repulsion. Upon dehydrogenation, the N atom moves 0.11 Å away from the surface, and the B moves 0.18 Å toward the surface, disrupting the bonding and the stability of the dimer. Therefore, dehydrogenation is most favored from opposite sides of the molecule as this increases the distance between dehydrogenated atoms, minimizing the disruption.

Dehydrogenation from a B or N from the *ortho* position alone cannot lead to trimerization due to the nearby hydrogen atoms preventing the B or N atoms of an incoming borazine molecule from occupying this position. Furthermore, as discussed in the dimerization pathway, dehydrogenation is required before the reaction. As dehydrogenation is preferred from adjacent hydrogen atoms on opposite sides of the molecule, additional borazine molecules are able to react with the B and N atoms in the *meta* positions of the molecule following a similar mechanism to dimerization. As shown in Section 3.1

Borazine molecules with adjacent dehydrogenated N–B–N atoms, dominate the surface at temperatures up to 700 K. Therefore, as illustrated in Figure 9, these are the most likely dimerization products with an *E*_r_ of −8.07 eV. The species has two exposed *ortho* nitrogens and an exposed *meta* nitrogen. As the energy differences between dehydrogenated dimers are small, the dehydrogenated structure formed is likely to be under kinetic control and will be determined by the availability of the partially dehydrogenated borazine species. Others of the same species are likely to attach to the *meta* nitrogen in trimerization. Following the trend of dehydrogenating adjacent hydrogen atoms, *meta* B atoms closest to the B–N bond are likely to become exposed in preference to the *para* B atoms furthest from the bond. As the *ortho* positions are inaccessible and the *para* positions are hydrogen‐terminated, we propose that the *meta* positions are the most likely sites for further polymerization. If the *ortho* sites were available, this could lead to the formation of surface clusters. Similarly, if *para* sites are available, this is likely to lead to line structures. The impact of dehydrogenation and steric selectivity is significant. Repeated reaction at the *meta* site will yield C‐shaped structures that will eventually combine into a nanopore, as discussed in Section 3.3.

### 3.3 Polymerisation of Borazine

Before we consider possible nanostructure formation, we look at several polymer structures formed out of three and four borazine molecules, respectively. **Figure** **10** shows polymer structures built from three molecules. There are four possible configurations which involve the combination of three borazine rings: **d** triangular cluster formation, **b** line formation with two borazine rings bonded on opposite sides of a central borazine molecule (*para* positions) and two C‐shaped structures that involve borazine molecules bonding to a central B or N atom (*meta* positions) as illustrated in **a** and **c**.

As the connectivity of each borazine ring is consistent, only the arrangement of the borazine molecules differentiates the polymer structures. The global minimum structure shown is a C‐shaped trimer with the B atoms on the central molecule facilitating bonding with the N atoms on the outer molecules (Figure 10a). A polymer where borazine molecules are bonded on either side of a central molecule to form a line is also plausible (Figure 10b), along with an analogous C‐shaped polymer where the central molecule bonds through N atoms (Figure 10c); these exhibit a Δ*E* of 0.004  and 0.108 eV, respectively. Additionally, three borazine molecules could form a cluster by loss of H_2_ with a Δ*E* of 0.175  eV. The small differences in relative energies of polymer structures suggest competition between the formation of line structures (Figure 10b) and C‐shaped structures (Figure 10a,c).

A combination of the structures **a** and **c** through the bonding of *meta* B and N atoms could form a nanopore with a central ring missing (Figure 12a). If either structure **b** or **d** dominates the surface, they are likely to polymerise to form linear (Figure 12b) structures or large hBN clusters, respectively. In order to form a cluster, dehydrogenation from the *ortho* positions on the same side of the dimer is required, which has been shown to be less favorable than dehydrogenation from opposite sides (Section 3.2). An additional borazine then needs to be approached from a more constrained and sterically hindered direction, which appears to be more difficult since the diffusion of borazine occurs by rotation around a stationary nitrogen atom. Hence, the probability of attachment from this site decreases, reducing the likelihood of cluster formation. Alternatively, any reaction is more likely to occur at the tail of the dimer at the more sterically accessible *para* or *meta* sites. As established in Section 3.2, dehydrogenation from the *meta* position appears more likely and has twice as many sites available per dimer (2 *para*, 4 *meta*). Therefore, the reaction of the *meta* sites of the dimer is more probable as they are more numerous, accessible and more easily dehydrogenated, which results in the formation of the “C‐shaped” trimers, as seen in Figure 10a,c.

**Figure 11:**
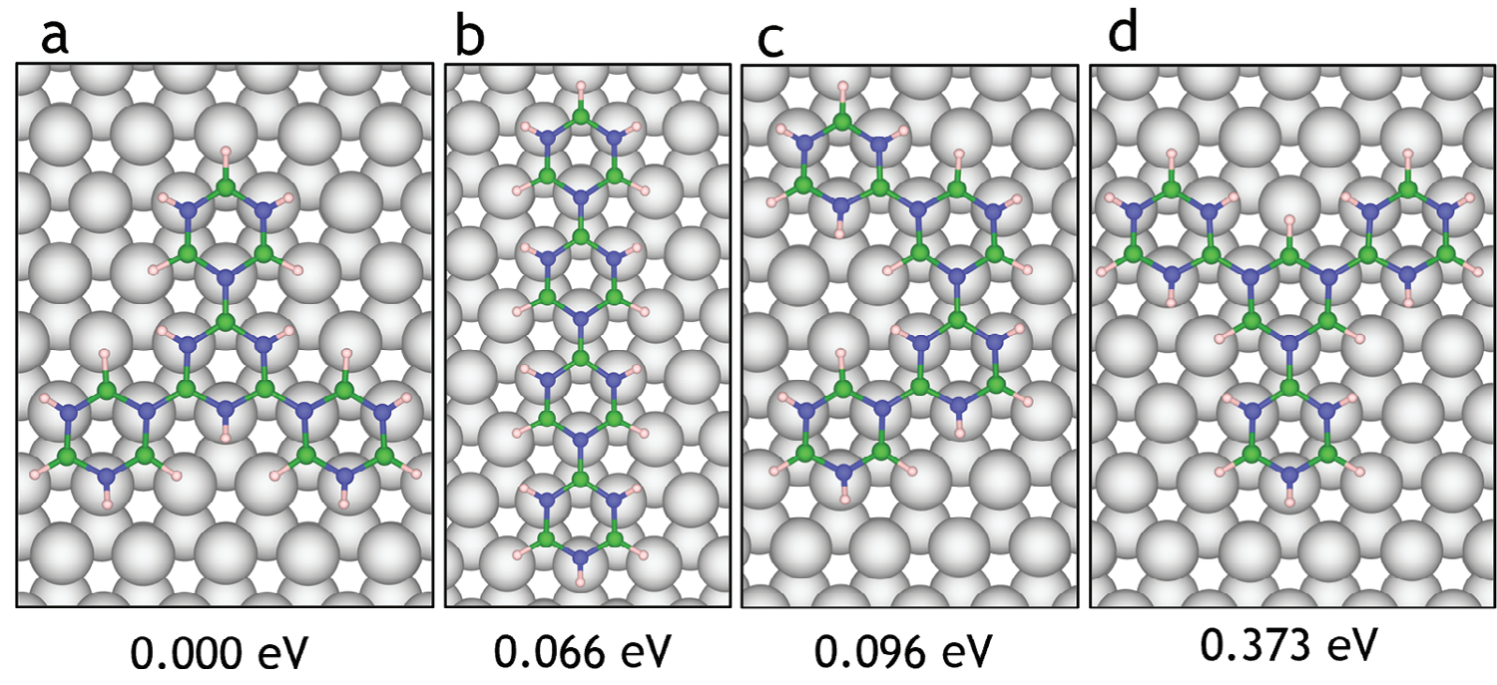
Relative energies for the formation of borazine tetramers with different configurations and increasing energy from left to right. (a) and (d) show polymerization through a single central borazine molecule at the *meta* position. In (a), the central borazine is connected via B atoms, and in (c), the central atom is connected via N atoms. (b) and (d) illustrate structures where each borazine molecule is connected to two others. In (b), each monomer is connected at the *para* position to form a ‘line’ structure. (d) shows a structure where each borazine molecule is connected in the meta position to form a “curve.” Here, (a), (c), and (d) bonded through *meta* positions can combine to form nanopores as illustrated in Figure 12a.

**Figure 12:**
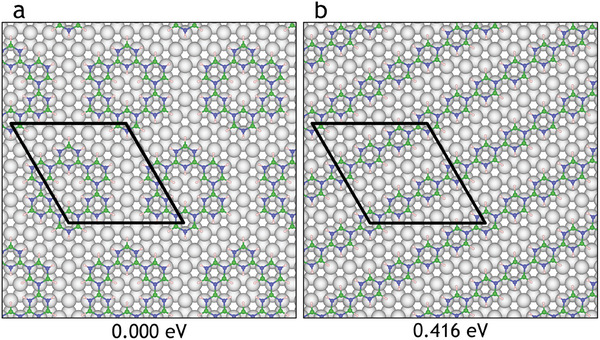
Relative energies of borazine polymer structures consisting of 6 monomer units per unit cell. (a) shows borazine monomers connected at the *meta* positions to form a ring, and (b) shows monomers connected at the *para* position to form a line.

Upon adding another borazine, a polymer consisting of four borazine rings is formed, with seven possible isomers. **Figure** **11** shows the four lowest energy structures with the global minimum structure occurring for three borazine molecules bonding to the three B atoms of a fourth central borazine molecule (Figure 11a). The isomer with borazine molecules arranged in a line (**b**) has a slightly higher energy than (**a**) followed by (**c**) with the borazine molecules arranged in a C‐shape, and (**d**) with the three outer borazine molecules bonding to the nitrogen atoms of the central borazine. As the connectivity is similar to that of the other isomers, the differences in energy are very small (ranging between 0 and 0.174 eV). It should be noted that structures (**a**), (**c**) and (**d**) involve the connection of borazine molecules at the *meta* position as opposed to (**b**) where the borazine molecules are connected through the *para* position. Repeated polymerization at the *meta* position can lead to nanopore formation (**Figure** **12**a). As there are more ways of arranging borazine molecules with connections at meta positions, the nanopores become statistically more likely than the formation of long linear structures. Structures (**a**), (**c**), and (**d**) could combine with one another to form nanoporous networks due to their complementary connectivity. Trimers are expected to follow dehydrogenation trends similar to monomers and dimers, with a preference for N dehydrogenation at *ortho* and *meta* positions, followed by B atoms adjacent to dehydrogenated N sites. Therefore, the trend of reaction and connection at the *meta* site appears to continue for larger polymer structures.

Figure 12 illustrates that upon filling a 6 × 6 unit cell with six borazine molecules, either an infinitely long line of *para*‐connected borazines can be created (**b**) or a ring of 6 *meta*‐connected borazine molecules (**a**). In both cases, the empty sites in‐between the structures can be connected to form a complete hBN layer, while the ring in (Figure 12a) additionally contains a hole in its center, which will need to be filled for a complete hBN layer. Thermodynamic stability affects structure formation, as the ring pattern is more stable by 0.42 eV than the line structure, the ring structure is thermodynamically favorable to form. For dimerization, the thermodynamic driving force for polymerization is the formation of B–N bonds. With more borazine rings added to the polymer structure, we thus expect the formation of larger structures. The exact structure which is formed will be governed by the CVD growth conditions, in particular the substrate temperature and borazine exposure, as they affect the kinetic mechanisms of dehydrogenation, diffusion and polymerization, which give rise to the formation of intermediate polymers during the formation of hBN.

For larger polymers (trimers and above), as shown in Figures 10, 11 and Figure 12, we can see the effect of substrate lattice mismatch. Outer borazine rings are pulled inwards by the strength of the B‐N bond, displacing the outer N and B atoms slightly from the Top and FCC sites. It is this effect that gives rise to a Moiré structure where 13 × 13 hBN unit cells coincide with 12 × 12 Ru(0001) unit cells,^[^ ^63^ ^]^ producing a corrugated sheet with valley and hill regions as has been observed in the literature.^[^ ^109^, ^110^, ^111^ ^]^ Due to the lateral size of this corrugated structure, we expect Moiré patterns to only occur on a domain size much larger than the supercells employed in our calculations.

**Figure 13:**
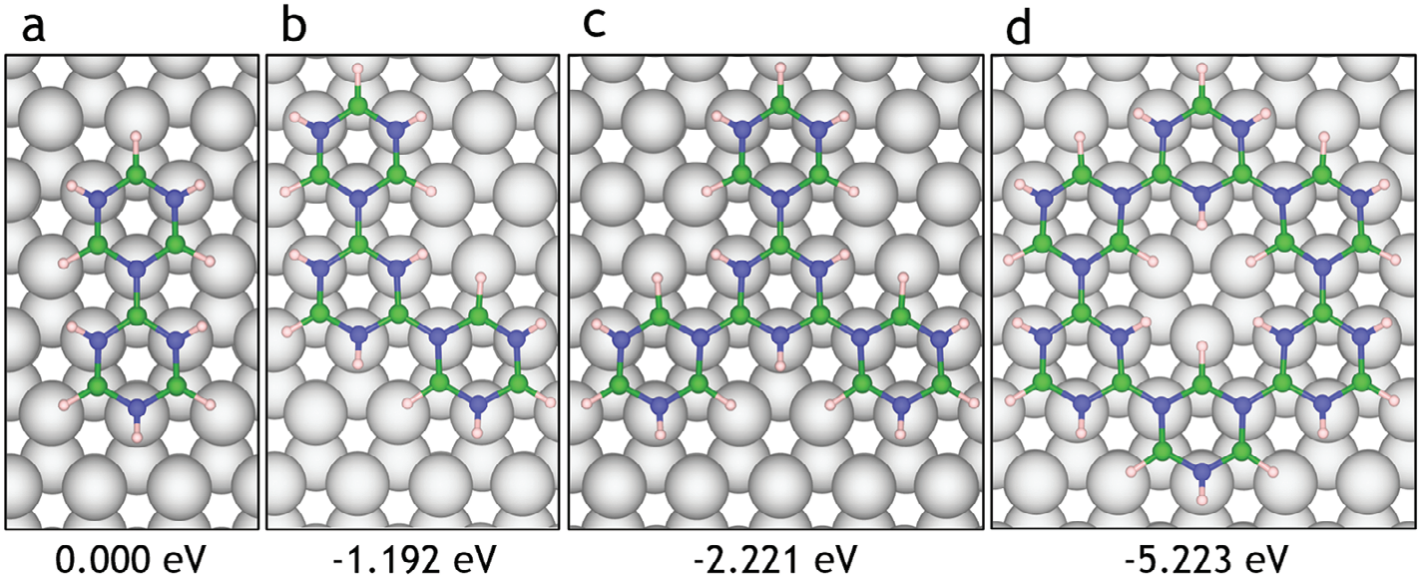
*E*_f_ for borazine polymers with 2–6 borazine molecules demonstrates that the formation of B‐N bonds is thermodynamically favoured, thus enabling the growth of borazine polymer structures, continuing with extensive nanostructures and eventually forming hBN. The formation energy decreases continuously from the borazine dimer (a), via a C‐shaped trimer bonded through *meta* B atoms (b), to a borazine tetramer bonded through *meta* B atoms (c), and finally a borazine ring polymer (d). Additionally, the *E*_f, m_ of polymers (a), (b), (c), and (d) are 0 eV, −0.397 eV, −0.555 eV, and −0.871 eV, respectively.

Finally, **Figure** **13** shows the most stable polymer structures for borazine polymers, according to *E*_f_, consisting of 2–6 borazine molecules.

In order to compare the energies of borazine polymer structures with differing numbers of borazine rings, we define a formation energy *E*_f_ relative to that of the adsorbed dimer and an appropriate number of isolated borazine molecules adsorbed on the surface:

(4) $$\begin{array}{cc} E_{f}= & E_{\mathrm{tot}}(x)+\beta E_{\mathrm{tot}}(H_{\mathrm{ads}})\\ & -E_{\mathrm{tot}}(\mathrm{DiB}Z_{\mathrm{ads}}+\mathrm{Ru})+\alpha E_{\mathrm{tot}}(BZ_{\mathrm{ads}})\; \end{array}$$

where *E*_tot_(*x*) is the total energy of the borazine polymer on the Ru(0001) surface, *E*_tot_(*DiBZ*_ads_ + *Ru*) is the total energy of the adsorbed dimer on Ru(0001), α is the number of additional borazine molecules added to create the polymer, *E*_tot_(*BZ*_ads_) is the energy of an adsorbed borazine molecule, β is the number of hydrogen atoms removed in bonding, and *E*_tot_(*H*_ads_) is the total energy of chemisorbed hydrogen. The *E*_f_ of the polymer can be calculated per number of monomers (*m*):

(5) $$E_{f,m}=E_{f}/m$$

The formation energy *E*_f_ for the most stable polymer structures in Figure 13 shows that as more monomers are added, *E*_f_ and *E*_f, m_ decrease.

These results are consistent with the findings in Section 3.2, where dimerization and polymerization are thermodynamically favorable. In general, the polymer becomes more stable as it grows in size and the number of B‐N bonds increases. Therefore, borazine structures at temperatures above 700 K are likely to continue via polymerization until adjacent structures connect and form an extended network of microporous intermediates before eventually filling the vacant sites and, thus, the formation of a complete hBN overlayer.

### 3.4 The Hydrogenated hBN Nanoporous Structure

Following the discussion of partially dehydrogenated borazine molecules (Section 3.1) in low‐temperature CVD conditions, we also considered the dehydrogenation of the nanopore structures by comparing the reaction energy *E*_r_ in a similar way to Equation 2 via:

(6) $$E_{r}=E_{\mathrm{tot}}\left(x\right)+\left(n+6\right)E_{\mathrm{tot}}\left(H_{\mathrm{ads}}\right)-E_{\mathrm{tot}}\left(Ru+2BZ\right)$$

where *E*_tot_(*x*) is the total energy of the nanopore species, *n* is the number of hydrogen atoms removed from the nanopore, *E*_tot_(*H*_ads_) is the total energy of chemisorbed hydrogen, and *E*_tot_(*Ru* + 2*BZ*) is the total energy of the isolated Ru surface and two isolated borazine molecules.

**Figure 14:**
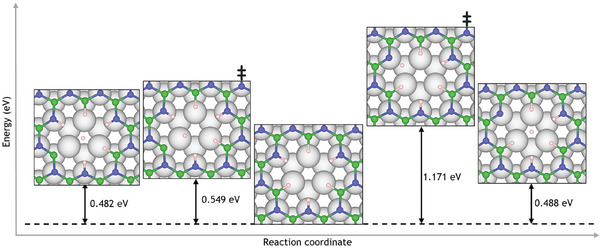
Nanopore dehydrogenation pathways to produce a dehydrogenated ring structure and an isolated H atom. Analogous to the single borazine molecule, B dehydrogenation is the kinetically favored product, whilst N dehydrogenation is thermodynamically favored: The nanopore can dehydrogenate by breaking a B–H or N–H bond with energy barriers of 0.549 and 1.171 eV, respectively, for the first dehydrogenation. On the other hand, Δ*E* for the reaction is +0.488 and +0.482 eV for B and N dehydrogenation, respectively.

While *E*_r_ of the dehydrogenated nanopore species can be found in Figure S9 (Supporting Information), **Figure** **14** shows the B and N dehydrogenation pathways from the nanopore, with the hydrogen moving to the HCP site above the Ru(0001) surface in the center of the nanopore, where it is expected to follow recombinative desorption as H_2_.^[^ ^115^ ^]^ Analogous to the isolated borazine molecule, N dehydrogenation is more favorable than B dehydrogenation. However, the difference in energy is less than 0.2 eV with reaction energies of −5.387  and −5.218  eV, respectively. Therefore, there is little thermodynamic preference for dehydrogenation from B or N atoms. Despite thermodynamic differences compared to dehydrogenation of the isolated borazine molecule, B‐dehydrogenation remains kinetically favorable with B and N transition state energy barriers of 0.549 and 1.171 eV, respectively, reflecting the relative strengths of B–H and N–H bonds as in Section 3.1.

Further dehydrogenation can then occur from either B or N sites, with 2N dehydrogenations continuing to be thermodynamically favourable, having an *E*_r_ of −4.220  eV, compared to 2B dehydrogenation with an *E*_r_ of −4.133  eV. Structures with B or N dehydrogenations are also possible with a reaction energy of –4.172 eV between the 2B and 2N dehydrogenation. Dehydrogenation from B continues to be kinetically favored, with an average barrier of 0.897 eV compared to 1.450 eV for N dehydrogenation. When examining the removal of three hydrogen atoms, the most favorable structure occurs with 2N and 1B dehydrogenation, resulting in an *E*_r_ of −3.004 eV, whereas the least favourable corresponds to only B dehydrogenations with an *E*_r_ of −2.808  eV. The slight preference for 2N and 1B dehydrogenated species relative to 3N (*E*_r_ of −2.98  eV) is likely due to a preference to dehydrogenate from adjacent sites, as discussed in Sections 3.1 and 3.2. Since a larger distance now separates the sites and for additional connections, the stabilizing effect is less pronounced, only becoming significant after three dehydrogenations. Continuing to the removal of four hydrogen atoms, the trend where N destinations are preferred is restored, with 4N and 4B dehydrogenation having an *E*_r_ of −1.743  and −1.459  eV, respectively. The mechanism continues until there is only one remaining hydrogen with an *E*_r_ of −0.326  and −0.107  eV for 5N and 5B, respectively.

Finally, complete dehydrogenation of the nanopore results in a structure with an *E*_r_ of +1.251 eV. A positive value for *E*_r_ indicates that the fully dehydrogenated structure is less thermodynamically stable than borazine monomers. All other fully and partially hydrogenated structures have negative values for *E*_r_, suggesting that the nonporous intermediate is likely to be partially hydrogenated during low‐temperature (700–900 K) hBN growth, which aligns with experimental findings.^[^ ^66^, ^67^, ^74^ ^]^

In order to complete the formation of a complete hBN overlayer, further borazine needs to react at the HCP site, in the center of the nanopore, and both the borazine and the nanopore need to be dehydrogenated. However, there are kinetic barriers for the hBN formation, considering specifically reduced access for incoming borzine molecules to surface sites necessary for the dehydrogenation. Furthermore, for borazine to dehydrogenate and form B–N bonds, conformational changes will be required for the molecule to fit inside the nanopore, thus requiring a higher energy barrier to complete the layer. Therefore, kinetic and thermodynamic factors inhibit the formation of hBN at lower temperatures, allowing the nanopore to be detected as an intermediate structure in experiments.^[^ ^63^ ^]^

### 3.5 Applicability To Other Substrates

The borazine‐based growth mechanism described in this work could extend to other low‐index transition metals due to borazine's structural and behavioral similarities. Substrates where hBN adopts a stable configuration with nitrogen occupying the N_TOP_ site (such as Ni(111), Cu(111), Rh(111), and Co(0001))^[^ ^57^, ^99^ ^]^ are promising additional candidates for the proposed growth mechanism. On these surfaces, borazine may self‐assemble into nanostructures due to diffusion between stable N_TOP_B_HCP_ and N_TOP_B_FCC_ sites, formed by partially dehydrogenated borazine molecules.

To assess the feasibility of this mechanism on other substrates, the behavior of borazine on Ni(111) was evaluated, with results presented in Figure S10 (Supporting Information). Similar to Ru(0001), the stable adsorption configurations on Ni(111) are N_TOP_B_HCP_ and N_TOP_B_FCC_, with an *E*_ads_ of −3.13  and −3.09  eV, respectively. Adsorption of nitrogen at the HCP and FCC sites results in *E*_ads_ values of −1.40  and −1.41  eV, respectively. Combined rotation and translation between FCC and HCP sites, achieved by pivoting around a stationary N_TOP_ site, has an energy barrier of 1.38 eV; in contrast, translation between FCC sites without rotation has a higher barrier of 1.87 eV. Initial dehydrogenation of borazine through breaking a B–H or N–H bond occurs with energy barriers of 0.81 and 1.11 eV, respectively, with adjacent deprotonation resulting in the most stable structures. The comparable dehydrogenation and diffusion barriers, along with similar relative site stabilities between Ru(0001) and Ni(111), the presented mechanism would also be applicable to describe the growth of hBN on Ni(111). Furthermore, the lower diffusion barrier on Ni(111) suggest polymerization and nanostructure formation at lower temperatures, with dehydrogenation and polymerization occurring within a narrower temperature range. These findings align with results from temperature‐programmed X‐ray photoelectron spectroscopy measurements of borazine deposited on Ni(111), where peaks attributed to disordered and defective hBN appear at temperatures exceeding 300 K, followed by the formation of hBN at temperatures above 600 K.^[^ ^112^ ^]^ The results thus indicate a significant temperature dependence in the formation of hBN, demonstrating consistent behavior of borazine on both Ni(111) and Ru(0001) surfaces, indicating that additional transition metals may exhibit a similar reaction mechanism.

In that context, it should be mentioned that previous theoretical works have also offered crucial insights into the high‐temperature growth mechanism of hBN on other low‐index transition metal surfaces. DFT and molecular dynamics studies using elemental B and N on a Ni substrate showed that hBN growth begins with the formation of linear BN chains that evolve into branched structures before forming hexagonal lattices and eventually propagating into larger hBN domains.^[^ ^76^ ^]^ Kinetic Wulff constructions have shown that the shape of hBN domains (triangles, truncated triangles, or hexagons) on Ni and Cu is dependent on growth kinetics controlled by the balance of B and N atoms.^[^ ^77^ ^]^ Concurrently, the suppression of defects in the hBN layer occurs due to the unfavorable B−B and N−N bond formation, leading to a uniform atomic structure as corroborated by molecular dynamics simulations using ReaxFF forcefields.^[^ ^78^, ^113^ ^]^ Furthermore, the quality and growth rate of hBN have been shown to depend on the roughness and presence of defects of the Ni substrate, as investigated through reactive force field molecular dynamics simulations.^[^ ^79^ ^]^ Finally, *ab initio* molecular dynamics simulations at 1500 K have shown that the pyrolytic decomposition of ammonia borane on Ni(111) proceeds via surface‐catalyzed reactions, producing BNH and BN dimers as key building units for hBN growth, whereas gas‐phase reactions encounter significantly higher energy barriers.^[^ ^80^ ^]^ These findings are most relevant to high‐temperature hBN growth using an alternative precursor gas instead of borazine.

## 4 Conclusion

In summary, we have presented a detailed microkinetic model for the synthesis of hBN on Ru(0001) from a borazine precursor. We follow the entire growth sequence, from initial borazine adsorption to subsequent dimerization, polymerization, and nanopore formation. Using density functional theory and microkinetic modeling, we have developed a model that accurately predicts both the abundance of and transformations between species across the entire relevant temperature regime, from 150 to 1500 K. The predictability of the model is demonstrated by excellent agreement with observations in experimental studies.^[^ ^57^, ^63^, ^67^, ^74^, ^108^ ^]^ These findings not only provide fundamental insights into the growth of hBN but also offer practical guidance for the synthesis of high‐quality hBN layers. Moreover, as indicated by initial calculations on Ni(111) where borazine exhibits similar behavior as on Ru(0001), the determined growth mechanism may also apply to other low‐index transition metal surfaces due to similarities of the borazine‐surface interaction mechanism.

In addition to providing a better understanding of the hBN growth mechanism in epitaxial CVD, we have further illustrated the crucial role of the precursor hydrogens in the process. The selectivity of BN species for specific adsorption sites (N_TOP_B_HCP_ and N_TOP_B_FCC_) consequently impacts the following surface diffusion path of the adsorbed borazine precursor. We further demonstrate that it is energetically favorable for the borazine precursor to undergo consecutive dehydrogenation, favoring the removal of hydrogen atoms from adjacent sites—a route which has not been considered in previous studies. The mentioned selectivity, coupled with limited diffusion, restricts dimerization to specific isomers with exposed *meta* sites, and the isomers further facilitate the formation of C‐shaped polymers and nanopore structures. Throughout the growth mechanism, partial rather than complete dehydrogenation and the sequence of dehydrogenation play a vital role in limiting the available sites for polymerization.

Following the sequence and energetics of these intermediate structures may create opportunities for a range of hBN‐based nanomaterials with varied structures and properties. In light of the recent experimental realisation of nanographenes in CVD growth,^[^ ^65^ ^]^ similar strategies should clearly be applicable to hBN nanostructures and nanopores but require further experimental research and studies for its realization. Further theoretical modelling approaches could include large‐scale molecular dynamics simulations employing classical or machine‐learned potentials to refine the predictions of the microkinetic model. As hBN growth is influenced by several parameters, including precursor, substrate, pressure and temperature, future modeling may greatly benefit from machine learning approaches.

## Conflict of Interest

The authors declare no conflict of interest.

## Author Contributions

A.P. conceived the presented idea, performed the DFT simulations and wrote the manuscript under the guidance of M.S.N.X. provided expertise in calculating vibrational properties and microkinetic modeling. A.T. provided expertise in surface chemistry of 2D materials growth and designed additional figures. All authors contributed to the discussion and interpretation of the results, edited the manuscript and approved the final version of the manuscript.

## Supporting information

Supporting Information

Supplemental Video 1

## Data Availability

The data that support the findings of this study are openly available in University of Surrey at https://doi.org/10.15126/surreydata.901161, reference number 901161.
