# Supplementary material for: Unravelling the Epitaxial Growth Mechanism of Hexagonal and Nanoporous Boron Nitride: A First‐Principles Microkinetic Model
Source: Small. 2025 Jan 5;21(10):2405404. doi: 10.1002/smll.202405404 (PMC11899527; doi:10.1002/smll.202405404)
Supplement: Supplementary file 1 — Supporting Information [file SMLL-21-2405404-s001.pdf]

NANO · MICRO
small

Supporting Information

for *Small*, DOI 10.1002/smll.202405404

Unravelling the Epitaxial Growth Mechanism of Hexagonal and Nanoporous Boron Nitride: A First-Principles Microkinetic Model

Anthony J. R. Payne, Neubi F. Xavier Jr, Anton Tamtögl and Marco Sacchi**

Supplementary information for: Unraveling the epitaxial growth mechanism of hexagonal and nanoporous boron nitride: A first-principles microkinetic model.†

Anthony J. R. Payne[a], Neubi F. Xavier Jr[a], Anton Tamtögl[b] and, Marco Sacchi[a].

1 Supplementary Figures

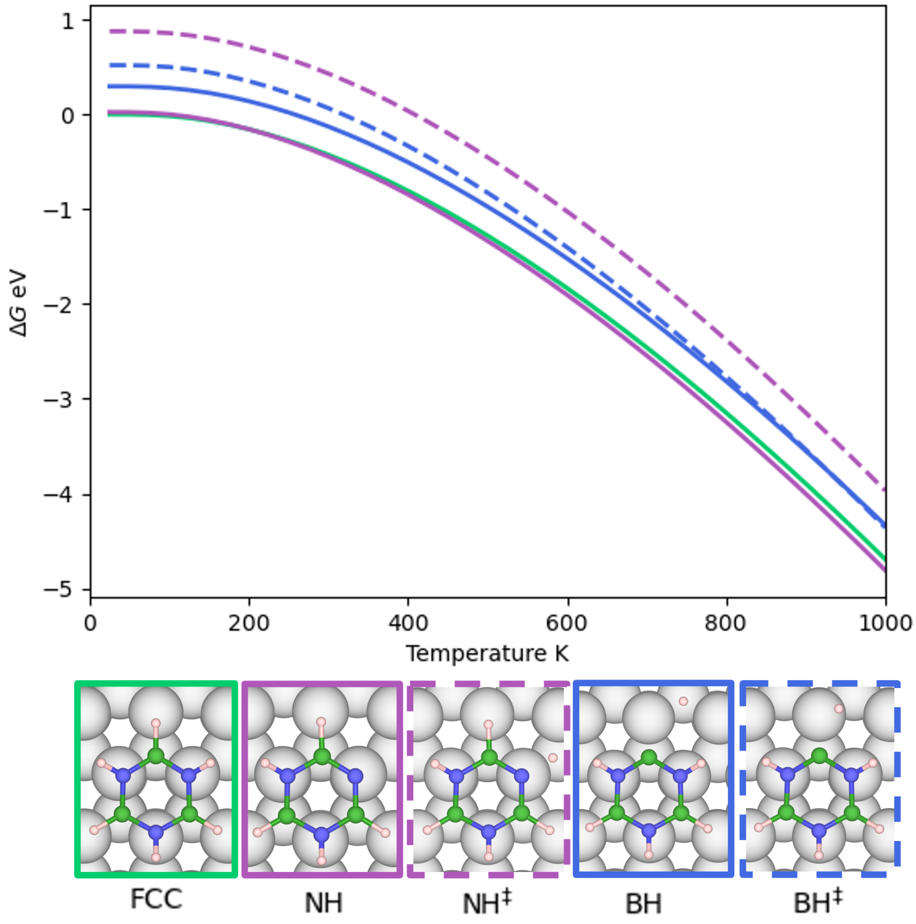

Fig. S1 Plot of G values for dehydrogenated borazine geometries and transition states relative to G of borazine in the FCC site. At around 250K, the relative G for N-dehydrogenation becomes negative and spontaneous above this temperature.

E-mail: m.sacchi@surrey.ac.uk; tamtoegl@tugraz.at
[a] School of Chemistry and Chemical Engineering, University of Surrey, Guildford, GU2 7XH, UK
[b] Institute of Experimental Physics, Graz University of Technology, Graz, Austria

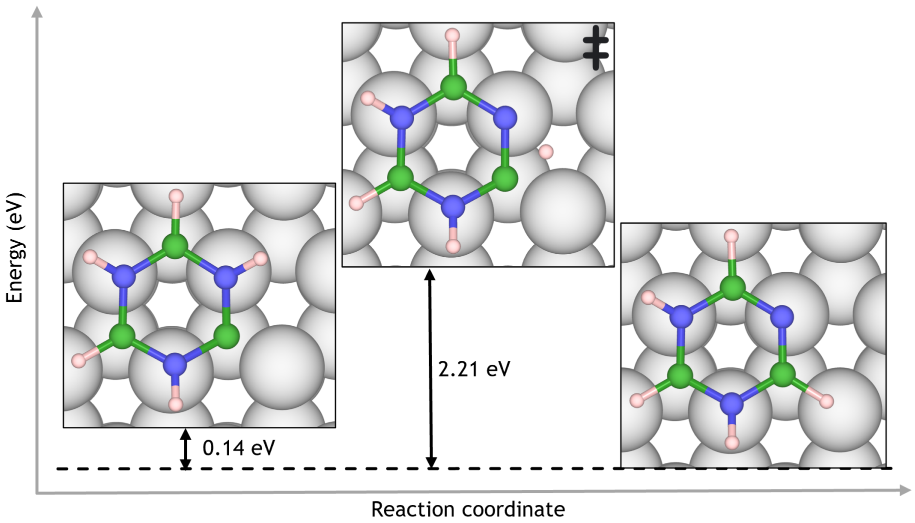

Fig. S2 Transfer of Hydrogen from N to B for a $B_3N_3H_5$ species proceeds through a barrier of 2.2 eV with a δH of -0.14 eV

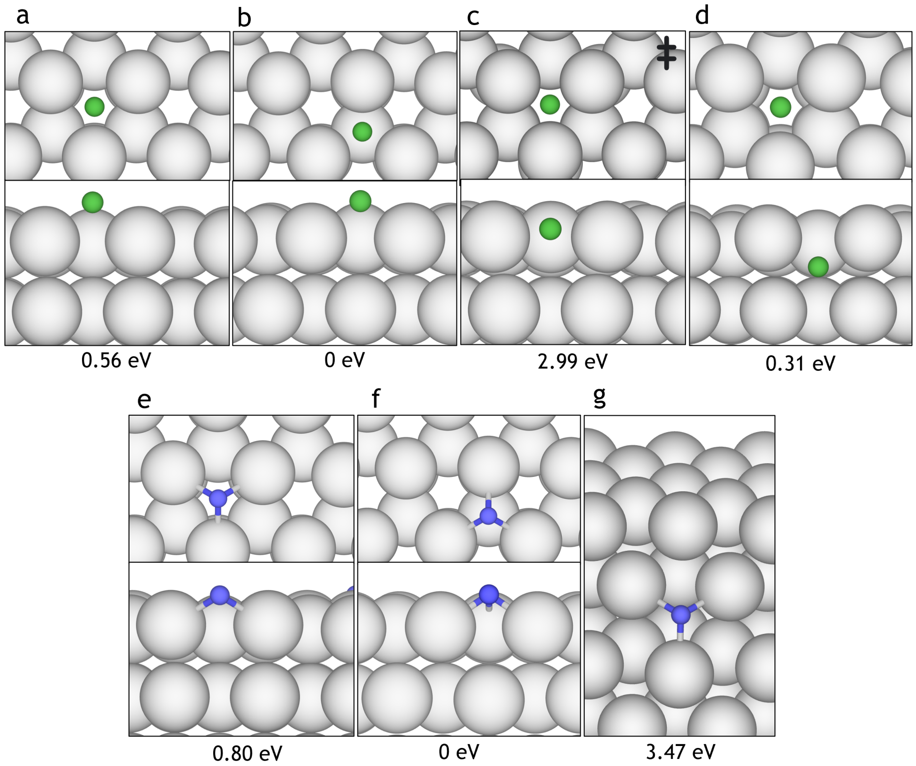

Fig. S3 Relative energies of an isolated Boron adatom in (a) FCC site (b) HCP site (c) transition state between HCP site and intercalation site (d) intercalation site below first Ru(0001) layer. Relative energies of isolated Nitrogen adatom in (e) FCC site (f) HCP site (e) in intercalation site below second Ru(0001) layer. All attempts to find a stable intercalation site for Nitrogen resulted in the Nitrogen returning to the FCC site or the HCP surface sites.

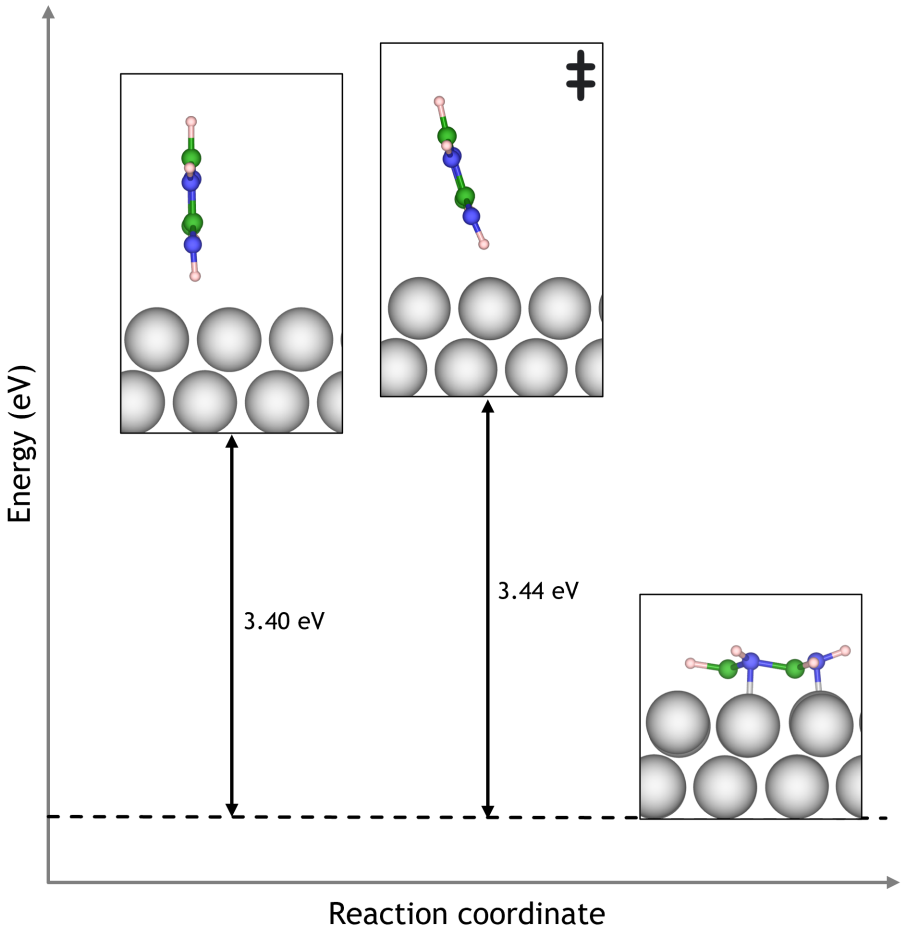

Fig. S4 Adsorption of borazine in a perpendicular orientation onto Ru(0001) through a transition state barrier of 0.04 eV. The ΔE of the reaction is 3.4 eV. Other orientations were considered, including borazine positioned parallel to the plain; however, no other physisorbed arrangement was found. The energy of this transition state barrier is so small it has been considered negligible.

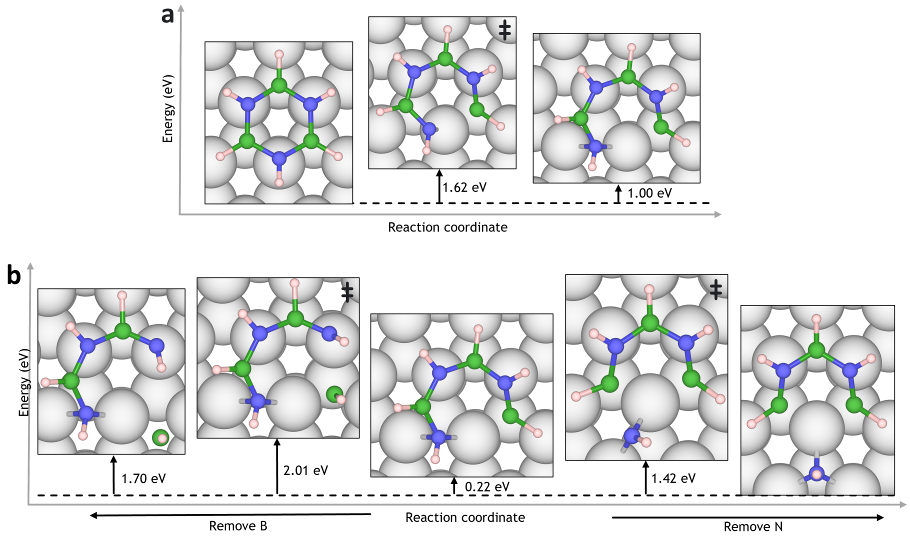

Fig. S5 The breaking of the borazine ($B_3N_3H_6$) ring is initiated via a two step process. (a) The breaking of a B-N bond. This is followed by (b) the breaking of a second bond, resulting in the removal of a B or N atom to the surface. Given the barriers involved in the reaction, we anticipate that ring breaking likely occurs after deprotonation.

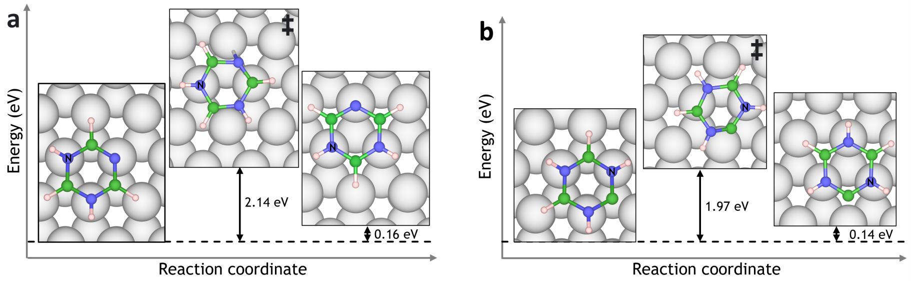

Fig. S6 Migration of partially dehydrogenated borazine species ($B_3N_3H_5$). (a) and (b) show the migration of B and N deprotonated borazine molecules, respectively on a Ru(0001) suraface. These migrations occur by rotation around a stationary nitrogen and with a similar energy barrier to the migration of borazine ($B_3N_3H_6$) as shown in figure 2 of the main text.

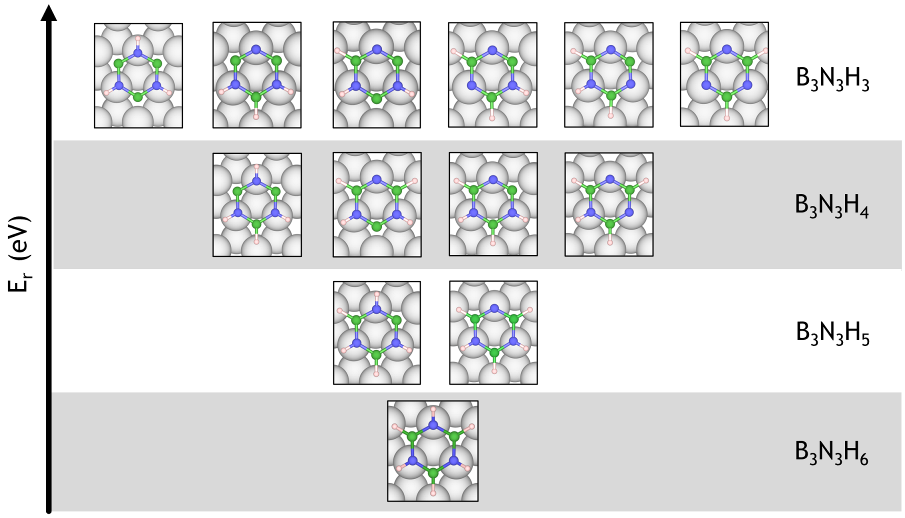

n in $B_3N_3H_n$	$E_r = E_{tot}(x) + nE_{tot}(H_{ads}) - E_{tot}(Ru + BZ)$					
3	-2.66	-2.87	-2.87	2.83	-2.86	-2.73
4	-3.13	-3.25		-3.29		-3.29
5	-3.70			-3.58		
6	-4.00					

Fig. S7 Reaction energies for dehydrogenated borazine structures ($B_3N_3H_n$) on HCP sites. The HCP deprotonated borazines have a higher E_r of 0.16 eV compared to the FCC equivalent.

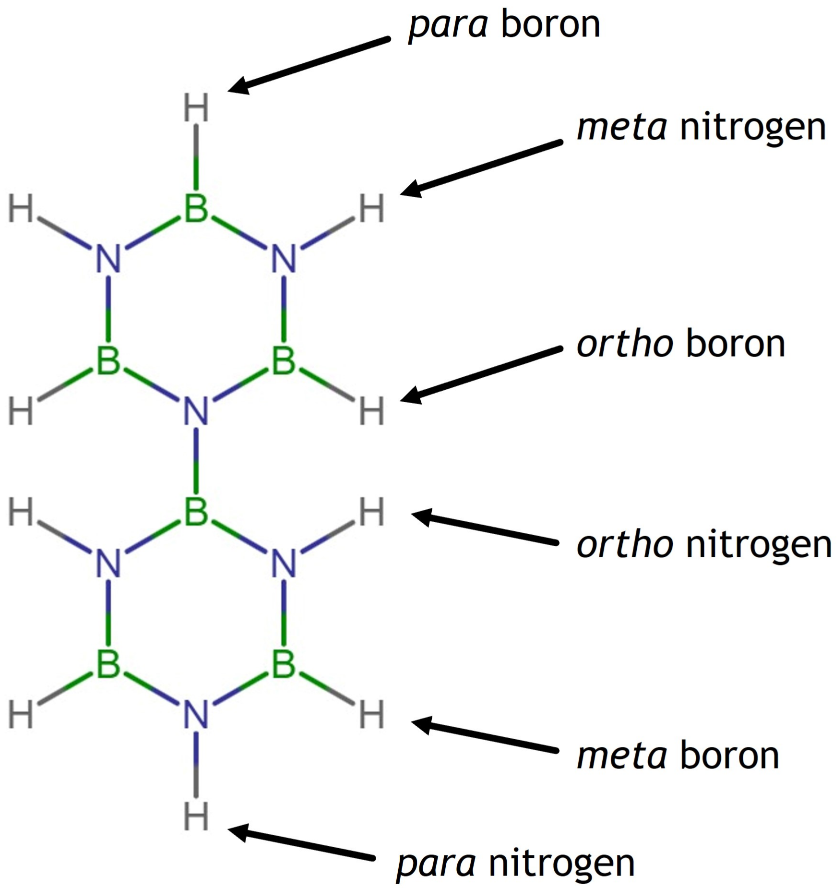

Fig. S8 Adoption of the *ortho*, *meta*, and *para* naming convection commonly used for substituted benzene rings applied to a borazine dimer.

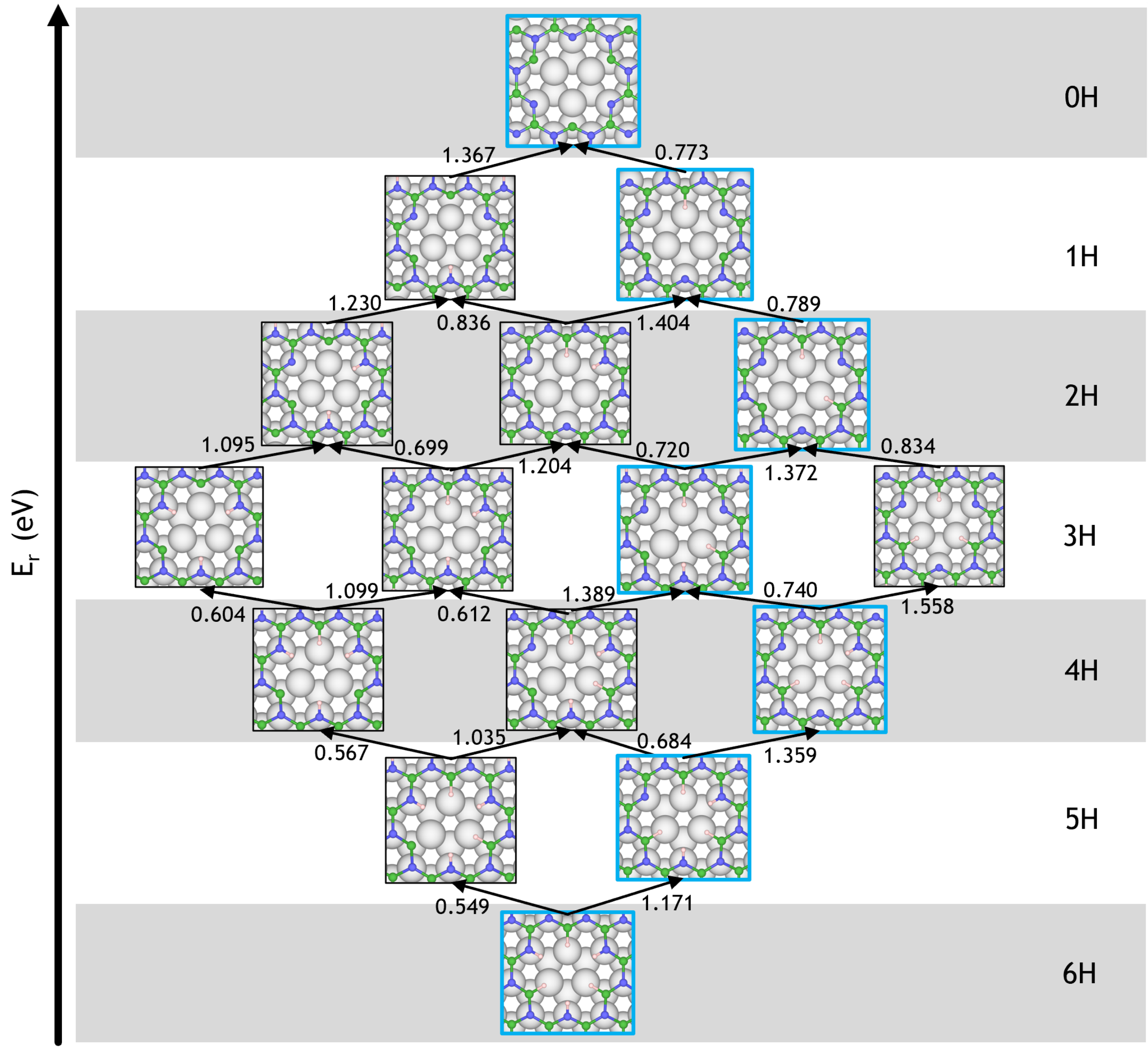

<table>
<tr><td colspan="12">1.251</td></tr>
<tr><td colspan="6">-0.107</td><td colspan="6">-0.326</td></tr>
<tr><td colspan="4">-1.459</td><td colspan="4">-1.616</td><td colspan="4">-1.743</td></tr>
<tr><td colspan="3">-2.808</td><td colspan="3">-2.952</td><td colspan="3">-3.004</td><td colspan="3">-2.980</td></tr>
<tr><td colspan="4">-4.113</td><td colspan="4">-4.172</td><td colspan="4">-4.220</td></tr>
<tr><td colspan="6">-5.218</td><td colspan="6">-5.387</td></tr>
<tr><td colspan="12">-6.286</td></tr>
</table>

Fig. S9 Reaction energies for dehydrogenated nanoporous hBN containing between 0 and 6 hydrogen atoms (0H - 6H), and transition state energy barriers are also shown. Reaction energies increase with increasing dehydrogenation, only becoming positive once all the hydrogen atoms have been removed, indicating a preference for borazine to remain isolated rather than bind and dehydrogenate completely. There is a general trend where N dehydrogenation is preferred. Transition state energies to dehydrogenate B are consistently lower (average 0.68 eV) compared to dehydrogenating from N (average 1.27 eV).

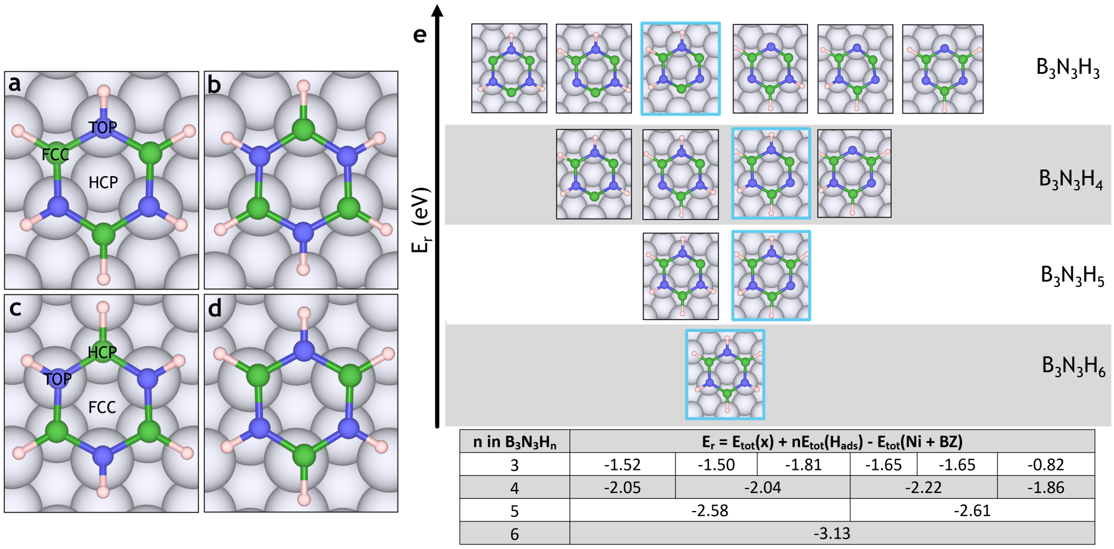

n in $B_3N_3H_n$	$E_r = E_{tot}(x) + nE_{tot}(H_{ads}) - E_{tot}(Ni + BZ)$					
3	-1.52	-1.50	-1.81	-1.65	-1.65	-0.82
4	-2.05	-2.04		-2.22		-1.86
5	-2.58			-2.61		
6	-3.13					

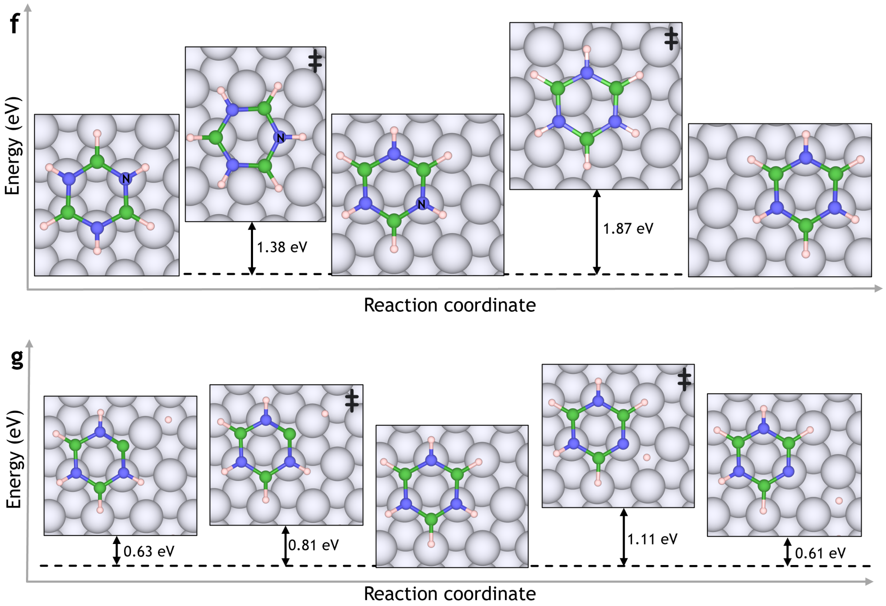

Fig. S10 Adsorption sites of Borazine on Ni(0001) (a) Borazine centred on the HCP site with N_{TOP} and B_{FCC} (E_{ads} -3.13 eV) (b) Borazine centred on the FCC site with N_{FCC} and B_{TOP} (E_{ads} -1.40 eV) (c) Borazine centred on the FCC site with N_{TOP} and B_{HCP} (E_{ads} -3.09 eV) (d) Borazine centred on the HCP site with N_{HCP} and B_{TOP} (E_{ads} -1.41 eV). Energetically, only the adsorption configurations (a) and (c) are likely. (e) Reaction energies for consecutive dehydrogenation from the complete borazine molecule to $B_3N_3H_3$ on Ni(111) with the most stable configuration for each stoichiometry highlighted with a blue border. (f) Diffusion pathways of borazine on Ru(0001). Rotation and translation between FCC and HCP sites occour via pivoting around a stationary N_{TOP} site (labelled N) with an energy barrier of 1.38 eV. Translation between FCC sites without rotation exhibits an energy barrier of 1.87 eV. (g) Dehydrogenation of borazine on Ni(111) by breaking a B-H or N-H bond with energy barriers of 0.81 eV and 1.11 eV, respectively.

2 Details of the microkinetic model

Kinetics simulations were performed in the MKMCXX to investigate the behaviour of borazine molecules on Ru(0001) surfaces.[1] Arrhenius parameters used in the model are shown in Tables S1-3. A simulation time of 10000 s was set to allow for steady-state convergence, and the maximum absolute and relative tolerances were set to 1x10E-16. Given the slow rate of reaction at low temperatures, specific temperatures below 390 K had failed to converge, and the simulation time was increased to 1E6 s; despite this at some temperatures below 400 K, complete, steady-state convergence was not achieved due to the slow nature of reactions at this temperature; therefore our coverage's may not be exact at these temperatures. However, our results largely match with experiments believe our results are still representative of the behaviour of borazine. A pressure of 5x10-8 mbar was applied to conform to experimental conditions. Since the dimerisation reaction requires borazine species to occupy adjacent HCP and FCC sites, with a marginal energy difference of 0.08 eV between these sites, we considered each of these sites to be equivalent in the model. Furthermore, the energy distinction between the deprotonated sites in HCP and FCC lattices is negligible as shown in Figure S7, so we expect minimal differences in Arrhenius parameters. The model was initialised with borazine in the gas phase. Surface sites for borazine molecules and atoms were treated separately (S-BZ and S) to reflect the larger size and low initial coverage of borazine. separately, As the dimerisation step is constrained by molecule migration and the migration of partially dehydrogenated borazines remains similar as shown in Figure S6, for model simplification, we assumed that identical Arrhenius parameters are applicable across the range of potential isomer combinations that can form a dimer. Given the adsorption energy of borazine (-4.08 eV), which further decreases on dehydrogenation,[2] we do not expect the dehydrogenated borazines to desorb $B_3N_3H_x$, so only consider the desorption of $B_3N_3H_x$. We justified the formation of C-shaped (-Ci) and line (-Li) based polymers based on the presence of available '-meta' or '-para' sites of the deprotonated borazine molecule. These simplifications allowed us to expand 20 unique reactions into 55 reaction representations to kinetically model the dehydrogenation, dimerisation, decomposition, adsorption, and absorption of borazine derivatives.

	Arrhenius coefficient (s^{-1})		Activation energy (eV)	
Label	Forward	Reverse	Forward	Reverse
$B_3N_3H_{6(g)}$ + S-BZ $\rightleftharpoons$ $B_3N_3H_{6(ads)}$	4.616100E+14	1.823500E+18	4.289040E+03	3.479645E+05
$B_3N_3H_{6(ads)}$ + S $\rightleftharpoons$ $B_3N_3H_5$-B + H_{ads}	3.352300E+13	2.144900E+13	5.372472E+04	1.348536E+04
$B_3N_3H_{6(ads)}$ + S $\rightleftharpoons$ $B_3N_3H_5$-N + H_{ads}	2.123200E+13	7.376500E+13	8.823234E+04	7.982856E+04
$B_3N_3H_5$-B $\rightleftharpoons$ $B_3N_3H_5$-N	8.786600E+13	6.082000E+12	1.855438E+05	2.097010E+05
$B_3N_3H_5$-B + S $\rightleftharpoons$ $B_3N_3H_4$-aBN + H_{ads}	4.205300E+13	3.048300E+13	9.253440E+04	8.110620E+04
$B_3N_3H_5$-N + S $\rightleftharpoons$ $B_3N_3H_4$-aBN + H_{ads}	6.163100E+12	6.454200E+13	5.520858E+04	9.517200E+03
$B_3N_3H_5$-B + S $\rightleftharpoons$ $B_3N_3H_4$-oBN + H_{ads}	7.633300E+13	2.061700E+14	9.678774E+04	8.380008E+04
$B_3N_3H_5$-N + S $\rightleftharpoons$ $B_3N_3H_4$-oBN + H_{ads}	5.283700E+12	2.259000E+12	5.845518E+04	1.184988E+04
$B_3N_3H_5$-B + S $\rightleftharpoons$ $B_3N_3H_4$-BN1 + H_{ads}	6.188600E+13	1.418600E+13	5.251344E+04	2.068584E+04
$B_3N_3H_5$-N + S $\rightleftharpoons$ $B_3N_3H_4$-NH1 + H_{ads}	1.277000E+12	7.839400E+13	1.051596E+05	7.438746E+04
$B_3N_3H_4$-aBN + S $\rightleftharpoons$ $B_3N_3H_3$-Nm + H_{ads}	1.119600E+14	7.765520E+13	5.165622E+04	2.230116E+04
$B_3N_3H_4$-aBN + S $\rightleftharpoons$ $B_3N_3H_3$-Bm + H_{ads}	1.288400E+16	2.366700E+14	1.007332E+05	7.529130E+04
$B_3N_3H_4$-aBN + S $\rightleftharpoons$ $B_3N_3H_3$-b + H_{ads}	1.586000E+16	8.494200E+15	1.045981E+05	6.943020E+04
$B_3N_3H_4$-BH1 + S $\rightleftharpoons$ $B_3N_3H_3$-BH0 + H_{ads}	1.193600E+14	1.366200E+14	5.551602E+04	3.247104E+04
$B_3N_3H_4$-BH1 + S $\rightleftharpoons$ $B_3N_3H_3$-NH0 + H_{ads}	3.368500E+15	5.338700E+15	1.187323E+05	6.569346E+04
$2H_{ads}$ $\rightleftharpoons$ $H_{2(g)}$ + 2S	5.663900E+14	2.349700E+11	1.172069E+05	2.035320E+03

Table S1 Parameters for the elementary reactions involved in the microkinetic modelling of the dehydrogenation of borazine ($B_3N_3H_6$ and dehydrogenated species $B_3N_3H_x$). S represents a free atom site on the surface, and S-BZ represents a free site for a $B_3N_3H_x$ species. $B_3N_3H_5$-B and $B_3N_3H_5$-N represent B and N deprotonated isomers, respectively. $B_3N_3H_4$-aBN and $B_3N_3H_4$-oBN represent $B_3N_3H_4$ isomers with deprotonation from adjacent B N sites and B N sites on opposite sides of the molecule. The suffixes -BH1 and -NH1 indicate isomers with only one remaining B-H or N-H bond, respectively. With suffixes: -Nm is adjacent dehydrogenation with 2 B and 1 N central N atom dehydrogenated, -Bm is adjacent dehydrogenation with 2 N and 1 B central B atom dehydrogenated, -b where there are two adjacent dehydrogenated atoms and a third separated by a bond (the isomer with 2 B and 1 N deprotonations is used), -BH0 and NH0 complete dehydrogenation of B atoms and N atoms respectively.

Label	Arrhenius coefficient (s^{-1})		Activation energy (eV)	
	Forward	Reverse	Forward	Reverse
$B_3N_3H_5$-B + $B_3N_3H_5$-B $\rightleftharpoons$ $2B_3N_3H_5$-Di_{ads}	3.760300E+13	1.163000E+14	2.179708E+05	3.879011E+05
$2B_3N_3H_4$-aBN $\rightleftharpoons$ $2B_3N_3H_4$-Ci	3.760300E+13	1.163000E+14	2.179708E+05	3.879011E+05
$2B_3N_3H_4$-oBN $\rightleftharpoons$ $2B_3N_3H_4$-Ci	3.760300E+13	1.163000E+14	2.179708E+05	3.879011E+05
$B_3N_3H_4$-oBN + $B_3N_3H_4$-aBN $\rightleftharpoons$ $2B_3N_3H_4$-Di	3.760300E+13	1.163000E+14	2.179708E+05	3.879011E+05
$B_3N_3H_4$-NH1 + $B_3N_3H_4$-BH1 $\rightleftharpoons$ $2B_3N_3H_4$-Ci	3.760300E+13	1.163000E+14	2.179708E+05	3.879011E+05
$B_3N_3H_4$-NH1 + $B_3N_3H_4$-aBN $\rightleftharpoons$ $2B_3N_3H_4$-Ci	3.760300E+13	1.163000E+14	2.179708E+05	3.879011E+05
$B_3N_3H_4$-BH1 + $B_3N_3H_4$-aBN $\rightleftharpoons$ $2B_3N_3H_4$-Ci	3.760300E+13	1.163000E+14	2.179708E+05	3.879011E+05
$B_3N_3H_4$-NH1 + $B_3N_3H_4$-oBN $\rightleftharpoons$ $2B_3N_3H_4$-Li	3.760300E+13	1.163000E+14	2.179708E+05	3.879011E+05
$B_3N_3H_4$-BH1 + $B_3N_3H_4$-oBN $\rightleftharpoons$ $2B_3N_3H_4$-Li	3.760300E+13	1.163000E+14	2.179708E+05	3.879011E+05
$2B_3N_3H_3$-Nm $\rightleftharpoons$ $2B_3N_3H_4$-Ci	3.760300E+13	1.163000E+14	2.179708E+05	3.879011E+05
$2B_3N_3H_3$-Bm $\rightleftharpoons$ $2B_3N_3H_4$-Ci	3.760300E+13	1.163000E+14	2.179708E+05	3.879011E+05
$2B_3N_3H_3$-b $\rightleftharpoons$ $2B_3N_3H_4$-Li	3.760300E+13	1.163000E+14	2.179708E+05	3.879011E+05
$B_3N_3H_3$-Bm + $B_3N_3H_3$-Nm $\rightleftharpoons$ $2B_3N_3H_4$-Ci	3.760300E+13	1.163000E+14	2.179708E+05	3.879011E+05
$B_3N_3H_3$-b + $B_3N_3H_3$-BH0 $\rightleftharpoons$ $2B_3N_3H_4$-Li	3.760300E+13	1.163000E+14	2.179708E+05	3.879011E+05
$B_3N_3H_3$-b + $B_3N_3H_3$-NH0 $\rightleftharpoons$ $2B_3N_3H_4$-Li	3.760300E+13	1.163000E+14	2.179708E+05	3.879011E+05
$B_3N_3H_3$-NM + $B_3N_3H_3$-b $\rightleftharpoons$ $2B_3N_3H_4$-Ci	3.760300E+13	1.163000E+14	2.179708E+05	3.879011E+05
$B_3N_3H_3$-BM + $B_3N_3H_3$-b $\rightleftharpoons$ $2B_3N_3H_4$-Ci	3.760300E+13	1.163000E+14	2.179708E+05	3.879011E+05
$B_3N_3H_3$-NH0 + $B_3N_3H_3$-BH0 $\rightleftharpoons$ $2B_3N_3H_4$-Ci	3.760300E+13	1.163000E+14	2.179708E+05	3.879011E+05
$B_3N_3H_3$-NH0 + $B_3N_3H_3$-Nm $\rightleftharpoons$ $2B_3N_3H_4$-Ci	3.760300E+13	1.163000E+14	2.179708E+05	3.879011E+05
$B_3N_3H_3$-NH0 + $B_3N_3H_3$-Bm $\rightleftharpoons$ $2B_3N_3H_4$-Ci	3.760300E+13	1.163000E+14	2.179708E+05	3.879011E+05
$B_3N_3H_3$-BH0 + $B_3N_3H_3$-Nm $\rightleftharpoons$ $2B_3N_3H_4$-Ci	3.760300E+13	1.163000E+14	2.179708E+05	3.879011E+05
$B_3N_3H_3$-BH0 + $B_3N_3H_3$-Bm $\rightleftharpoons$ $2B_3N_3H_4$-Ci	3.760300E+13	1.163000E+14	2.179708E+05	3.879011E+05

Table S2 Parameters for the elementary reactions involved in the microkinetic modelling of the dimerisation of borazine species ($B_3N_3H_x$. $B_3N_3H_x$-Li or-Ci represent $B_3N_3H_x$ isomers that are associated with linear or C-shaped polymers. The $B_3N_3H_x$-Di suffix represents the formation of a dimer that belongs to neither of these groups).

Label	Arrhenius coefficient (s^{-1})		Activation energy (eV)	
	Forward	Reverse	Forward	Reverse
$B_3N_3H_5$-N + S $\rightleftharpoons$ $B_3N_2H_5$ + N_{ads}	2.610300E+12	8.188600E+13	2.003030E+05	1.587503E+05
$B_3N_3H_5$-B + S $\rightleftharpoons$ $B_2N_3H_5$ + B_{ads}	8.180300E+14	3.447900E+13	4.408463E+05	1.810637E+05
$B_3N_3H_4$-aBN + S $\rightleftharpoons$ $B_3N_2H_4$ + N_{ads}	2.610300E+12	8.188600E+13	2.003030E+05	1.587503E+05
$B_3N_3H_4$-aBN + S $\rightleftharpoons$ $B_2N_3H_4$ + B_{ads}	8.180300E+14	3.447900E+13	4.408463E+05	1.810637E+05
$B_3N_3H_4$-oBN + S $\rightleftharpoons$ $B_3N_2H_4$ + N_{ads}	2.610300E+12	8.188600E+13	2.003030E+05	1.587503E+05
$B_3N_3H_4$-oBN + S $\rightleftharpoons$ $B_2N_3H_4$ + B_{ads}	8.180300E+14	3.447900E+13	4.408463E+05	1.810637E+05
$B_3N_3H_4$-NH1 + S $\rightleftharpoons$ $B_3N_2H_4$ + N_{ads}	2.610300E+12	8.188600E+13	2.003030E+05	1.587503E+05
$B_3N_3H_4$-BH1 + S $\rightleftharpoons$ $B_2N_3H_4$ + B_{ads}	8.180300E+14	3.447900E+13	4.408463E+05	1.810637E+05
$B_3N_3H_3$-Nm + S $\rightleftharpoons$ $B_3N_2H_3$ + N_{ads}	2.610300E+12	8.188600E+13	2.003030E+05	1.587503E+05
$B_3N_3H_3$-Nm + S $\rightleftharpoons$ $B_2N_3H_3$ + B_{ads}	8.180300E+14	3.447900E+13	4.408463E+05	1.810637E+05
$B_3N_3H_3$-Bm + S $\rightleftharpoons$ $B_3N_2H_3$ + N_{ads}	2.610300E+12	8.188600E+13	2.003030E+05	1.587503E+05
$B_3N_3H_3$-Bm + S $\rightleftharpoons$ $B_2N_3H_3$ + B_{ads}	8.180300E+14	3.447900E+13	4.408463E+05	1.810637E+05
$B_3N_3H_3$-b + S $\rightleftharpoons$ $B_3N_2H_3$ + N_{ads}	2.610300E+12	8.188600E+13	2.003030E+05	1.587503E+05
$B_3N_3H_3$-b + S $\rightleftharpoons$ $B_2N_3H_3$ + B_{ads}	8.180300E+14	3.447900E+13	4.408463E+05	1.810637E+05
$B_3N_3H_3$-NH0 + S $\rightleftharpoons$ $B_3N_2H_3$ + N_{ads}	2.610300E+12	8.188600E+13	2.003030E+05	1.587503E+05
$B_3N_3H_3$-BH0 + S $\rightleftharpoons$ $B_2N_3H_3$ + B_{ads}	8.180300E+14	3.447900E+13	4.408463E+05	1.810637E+05
B_{ads} $\rightleftharpoons$ B_{bulk} + S	3.907700E+12	8.454300E+12	1.472470E+05	1.159796E+05

Table S3 Parameters for the elementary reactions involved in the microkinetic modelling to break the borazine ring into constituent atoms. The Notation for each species gives the number of atoms in a molecule. For example, $B_3N_2H_5$ contains 3 Boron 2 Nitrogen and 5 Hydrogen atoms.

References

1 I. A. Filot, R. A. Van Santen, E. J. Hensen, *Angewandte Chemie - International Edition* **2014**, *53* (47), 12746–12750.

2 A. Ruckhofer, M. Sacchi, A. J. R. J. R. Payne, A. P. Jardine, W. E. Ernst, N. Avidor, A. Tamtögl, *Nanoscale Horizons* **2022**, *7* (11), 1388–1396.
